# Enhanced MOF performance in chromium(vi) removal from water using tailored MOF-polymer composites

**DOI:** 10.1039/d5sc05812k

**Published:** 2025-10-14

**Authors:** Timo M. O. Felder, Wei Shi, Daniel T. Sun, Till Schertenleib, Emad Oveisi, Jordi Espín, Wendy L. Queen

**Affiliations:** a Institute of Chemical Sciences and Engineering (ISIC), École Polytechnique, Fédérale de Lausanne (EPFL) CH-1950 Sion Switzerland wendy.queen@epfl.ch; b Research Center for Analytical Sciences, Department of Chemistry, College of Sciences, Northeastern University Shenyang 110819 China; c Interdisciplinary Center for Electron Microscopy (CIME), École Polytechnique Fédérale de Lausanne (EPFL) CH-1015 Lausanne Switzerland; d Sunchem Inc. 2300 4th St Berkeley CA 94710 USA

## Abstract

Functionalizing the internal and external surfaces of MOFs with polymers allows for tailor-made improvements in their performance for chemical separations. In this work, various MOF/polymer composites are screened for the extraction of Cr(vi) from water. One material, which consists of polyserotonin (PS) inserted into Fe-BTC (Fe-BTC/PS), outperformed other screened materials in acidic media (pH = 3). The material offers a maximum removal capacity of 106 mg g^−1^, which is approximately 10 times higher than that of the bare MOF Fe-BTC (9.8 mg g^−1^) at pH 3. The Cr(vi) extraction is achieved *via* a combined adsorption–reduction mechanism, which is driven by the highly porous MOF combined with a redox-active polymer. Furthermore, for the best-performing material, a protective external polymeric coating was applied which allowed Cr(vi) decontamination in an even more acidic medium (pH 2). In a similar fashion, the integration of polyserotonin into more acid-stable Cr/Zr-MOFs (MIL-101(Cr) and UiO-66(Zr)) showed improved removal of toxic Cr(vi) from acidic aqueous solutions. Finally, the Fe-BTC/PS composite was also able to reduce the Cr(vi) concentration in chromium-spiked real-world river water samples at neutral pH to levels below the WHO recommended guideline of 50 ppb with an adsorbent dosage of only 0.25 g L^−1^.

## Abstract 2

Functionalizing the internal and external surface of MOFs with polymers allows for tailor-made improvements in their performance for chemical separations.

## Graphical abstract

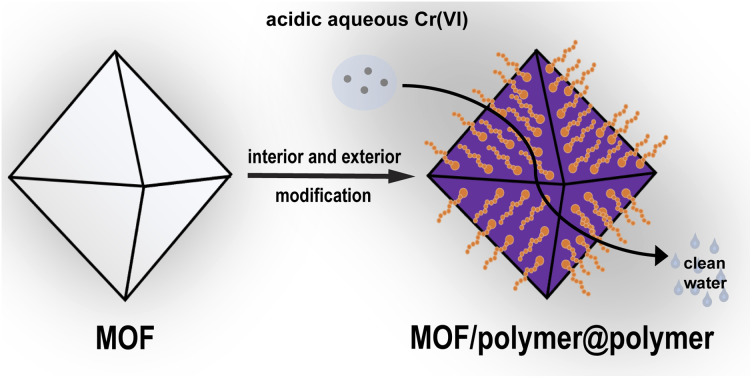

## Introduction

High porosity is one of the most sought-after material properties as it can boost diffusion, mass transfer, and adsorption capacities of targeted chemical species unlocking a number of important applications in sensing, drug delivery, catalysis, and/or separations. Notably, metal–organic frameworks (MOFs)^1^ have gained substantial attention due to several key characteristics including unprecedented internal surface areas and pore volumes,^2^ and high chemical tunability.^3–5^ Owing to the latter, MOF studies have reported over 90 000 distinct frameworks.^6,7^ Likewise, porous organic polymers are also an extensively investigated class of materials in the literature, revealing promise in various research areas including gas separation, catalysis, water treatment or biomedical applications.^8,9^ Notably, porous polymers are often constructed from pre-defined, rigid and/or contorted organic building blocks that inhibit space-efficient packing.^10^ While the use of such building blocks is effective to prepare porous polymers, there are still challenges to incorporate certain functionality into their backbone while maintaining porosity.^11^ Considering this, we and others have in recent years shown that impregnating MOFs with polymers can lead to highly porous composite materials having a host of benefits in liquid and gas separations.^12–18^ For example, in such composites, the porous MOF allows incoming guests to access the backbone of the otherwise non-porous polymer, while the high density of adsorption sites on the polymer backbone can drastically enhance adsorption capacities and selectivities relative to the parent MOF. Further, polymers were shown to also provide non-native functionality, like redox activity, or improved mechanical and chemical stability to MOFs.^15^

In previous work, we showed that the integration of redox-active polymers into MOF pores enhanced the removal of metal ions including Hg^2+^,^19^ Ag^+^,^20^ Pt,^21^ Pd^2+^,^22^ and Au^3+^ (ref. 18) from aqueous streams. Building on this, we hypothesize that this MOF/polymer chemistry could also be effectively applied to target other hazardous redox-active metals, such as Cr(vi). The highly toxic hexavalent chromium is a potent environmental pollutant that originates from both industrial activities, like stainless steel production, electroplating, and leather tanning industries,^23^ and natural geological processes.^24^ Cr(vi) compounds are classified as human carcinogens and exert additional genotoxic and oxidative stress effects, making even low-level exposure a significant public-health concern.^25,26^ Due to this risk, stringent drinking-water guidelines restrict chromium to tens of μg L^−1^ (WHO/EPA), with growing regulatory emphasis on specifically limiting Cr(vi).^23,27^ Consequently, technologies that can selectively capture Cr(vi) are of immediate practical importance for protection of drinking-water supplies and mitigation of industrial effluents.

In industrial settings, one often encounters acidic waste streams (pH < 3) with chromium speciation including HCrO_4_^−^ and HCr_2_0_7_^−^/Cr_2_O_7_^2−^ for Cr(vi) and Cr^3+^ ions for Cr(iii).^28^ The formation of dichromate species (*e.g.* Cr_2_O_7_^2−^) is both dependent on the pH and chromium concentration.^29,30^ Therefore, at dilute chromium concentrations and low pH (our experimental conditions), we expect that the dominant species for the highly toxic Cr(vi) are negatively charged ions (HCrO_4_^−^) whereas the less toxic Cr(iii) often forms a soluble chromium aqua complex ([Cr(H_2_O)_6_]^3+^).^28^ To target the extraction of HCrO_4_^−^, several structurally distinct MOFs were impregnated with varying redox-active polymers that possess a high density of Lewis base functionality on their backbone, which could adsorb and reduce Cr(vi) species to the less toxic Cr(iii) species. Therefore, the composites were subsequently assessed for the extraction of the highly toxic HCrO_4_^−^ from river water spiked with HCrO_4_^−^/CrO_4_^2^.

## Results and discussion

**Scheme 1:**
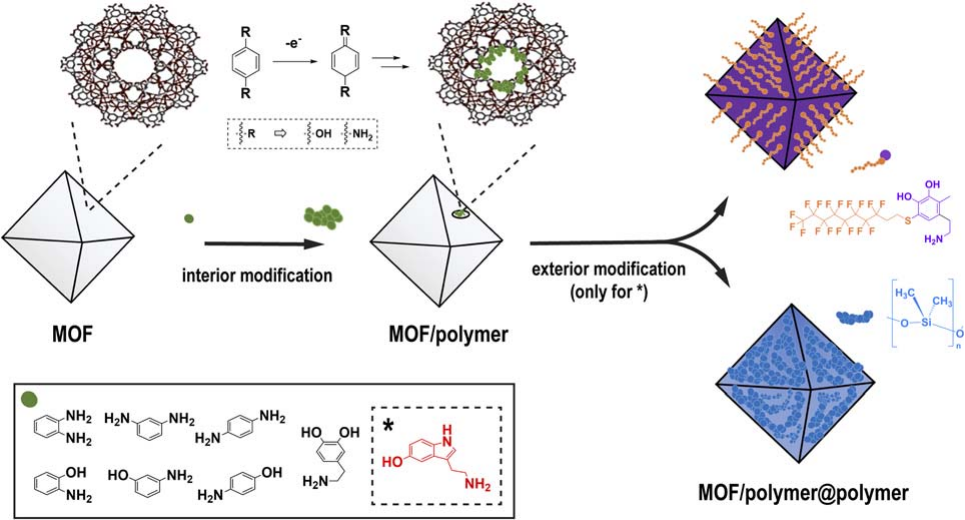
Schematic showing an overview of the two-step process used to decorate the internal and external surface of the MOFs with polymers.

To start, a series of new composites was synthesized using different monomeric building blocks and a MOF known as Fe-BTC as a porous support. The 3D network of Fe-BTC is composed of triangular Fe_3_O clusters coordinated to benzene-1,3,5-tricarboxylate linkers (BTC) – offering a high surface area (1556 m^2^ g^−1^) and mesoporous cages (∼25 Å and 29 Å) accessible through microporous windows (5.5 and 8.6 Å).^31^ The accessible Fe^3+^ sites, present on the internal surface of Fe-BTC, can oxidize amine (–NH_2_) or hydroxyl (–OH) groups on the aromatic monomers to imines (=NH) or quinones (=O) (Scheme 1). The oxidized molecules can subsequently react with other monomers, polymerizing inside the MOF pore. With this mechanism in mind, it was thought that the incorporation of polymers having subtle structural variations could readily be incorporated into the MOF, providing us with the opportunity to assess how fine structural changes might enhance performance in Cr(vi) extraction. For instance, isomers of aminophenol, including *o*-aminophenol, *m*-aminophenol, and *p*-aminophenol, share the same molecular formula, but form slightly different polymeric structures.^32^ Similar to this, isomers of phenylenediamine, *o*-phenylenediamine (*o*PDA), *m*-phenylenediamine (*m*PDA) and *p*-phenylenediamine (*p*PDA) as well as neurotransmitters dopamine (DA) and serotonin (S) were also incorporated into the MOF.

The *in situ* polymerization reactions were carried out using various methods previously reported by our group,^13,19^ allowing the introduction of polymeric aminophenol isomers, phenylenediamine isomers, serotonin, and dopamine in the MOF pores. The resulting eight MOF/polymer composites were denoted as Fe-BTC/P(*o*/*m*/*p*)AP, Fe-BTC/P(*o*/*m*/*p*)PDA, Fe-BTC/PS and Fe-BTC/PDA. In all cases, the crystalline phase of Fe-BTC is retained post-polymerization (Fig. S1–S4), and as expected, there is a reduction in Brunauer–Emmett–Teller (BET) surface area when compared to the parent MOF (Fig. S1–S4 and Table S1) owing to the polymer insertion inside the pores. Similar polymer loadings, in the range from 10 to 16 wt%, were found *via* combustion and thermogravimetric analysis (TGA) for Fe-BTC/PpAP, Fe-BTC/PpPDA, Fe-BTC/PS, and Fe-BTC/PDA, whereas significantly lower loadings (<2.3%) were observed for other polymers (Table S2).

**Fig. 1:**
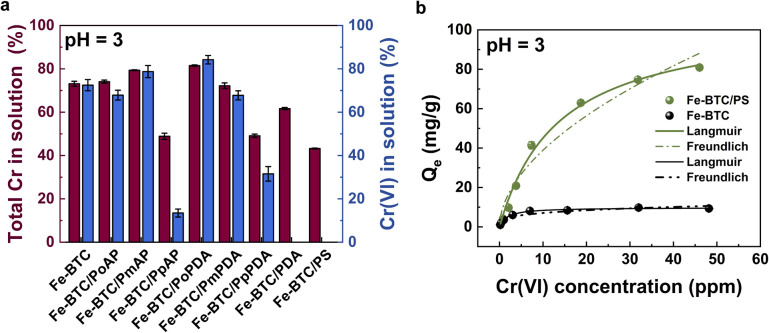
(a) Results of batch adsorption experiments in 10 ppm Cr(vi) solutions for all 8 synthesized MOF/polymer composites; red shows the % of total chromium in solution after 24 h of adsorption measured *via* ICP-OES and blue shows the % of Cr(vi) measured *via* UV-vis. (b) Cr(vi) adsorption isotherm for Fe-BTC (black) and Fe-BTC/PS (green) at pH 3.

Next, the performance of the composites was evaluated in acidic conditions. For this, all Fe-BTC/polymer composites were subjected to batch adsorption experiments in 10 ppm Cr(vi) solutions (Fig. 1a, adsorption capacities in Fig. S5). Although Fe-BTC/PDA, Fe-BTC/PpPDA, Fe-BTC/PpAP, and Fe-BTC/PS were all able to reduce toxic Cr(vi) to the less toxic Cr(iii) in solution based on UV-vis measurements of the chromium solution after adsorption (Fig. 1a), Fe-BTC/PS had the highest chromium capacity, indicating that it may be a better sorbent for the resulting Cr(iii)-containing species. Given its better performance, Fe-BTC/PS was selected for a more detailed study. In a screening for Cr(vi) extraction using different polymer loadings, an optimal loading of 12.8 wt.% polyserotonin was identified (Fig. S6). The material exhibited excellent crystallinity after polymerization (Fig. S1) and retained a high BET surface area of 1139 m^2^ g^−1^ (Table S1). The N 1s region of the X-ray Photoelectron Spectroscopy (XPS) data illustrates the presence of primary and secondary amines, which indicate successful integration of PS into the framework (Fig. S7). To prove that the polymer is homogeneously located throughout the porous template, Fe-BTC/PS particles were embedded in an epoxy resin and then serially sectioned into approximately 60 nm-thick slices (*via* ultramicrotomy) for electron microscopy investigations. Energy-Dispersive X-ray Spectroscopy analysis in Scanning Transmission Electron Microscopy (STEM-EDX) shows the distribution of different elements inside the crystallite. Nitrogen, a signature element of the polymer that is not present in the parent MOF structure, is indeed distributed throughout the composite's porous architecture (Fig. S8). Further, EDX line scans illustrate that the nitrogen counts terminate at the same position as the iron counts, confirming the polymer is inside Fe-BTC, rather than on the external surface (Fig. S8).

**Fig. 2:**
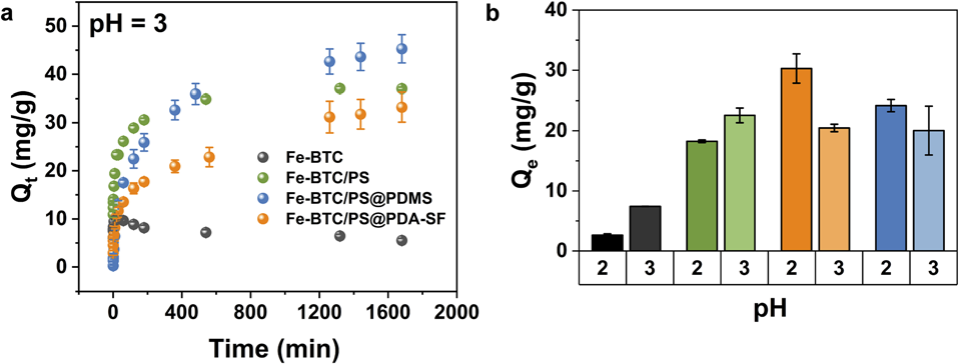
(a) The extraction of Cr(vi) species plotted as a function of time by Fe-BTC, Fe-BTC/PS, Fe-BTC/PS@PDA-SF, and Fe-BTC/PS@PDMS from aqueous acidic media (pH 3); (b) Cr(vi) batch adsorption experiments at pH 2 and 3 for Fe-BTC (black), Fe-BTC/PS (green), Fe-BTC/PS@PDA-SF (orange) and Fe-BTC/PS@PDMS (blue).

Next, a thorough study on HCrO_4_^−^ removal by Fe-BTC/PS from acidic media was carried out. The adsorption isotherm, fitted using the Langmuir model, revealed a maximum adsorption capacity of 106 mg g^−1^ for Fe-BTC/PS, which is around 10 times higher than that of the parent MOF (Fig. 1b and Table S3). Furthermore, after exposure to HCrO_4_^−^ solutions, STEM-EDX shows that chromium is distributed homogeneously throughout the MOF/polymer composite (Fig. S8) and XPS measurements indicate that all extracted Cr(vi) is reduced to the less toxic Cr(iii) (Fig. S9). Surprisingly, when tested in solutions containing [Cr(H_2_O)_6_]^3+^ the MOF and MOF/polymer composite have minimal extraction efficiency, further supporting a combined adsorption–reduction mechanism (Fig. S10). While future mechanistic studies would be interesting, we presume that the redox activity of the polymer (–OH, –NH–) and its metal-chelating moieties (–CH_2_CH_2_NH_2_) result in the effective adsorption and reduction of hexavalent chromium without the intermediate formation of [Cr(H_2_O)_6_]^3+^. Next, the kinetic properties of Fe-BTC and Fe-BTC/PS were evaluated and compared (Fig. 2a). While Fe-BTC/PS demonstrated a rapid removal rate (*k*_2_ = 1.8 × 10^−3^ g mg^−1^ min^−1^), as determined by fitting the data with the pseudo-second order kinetic model (Fig. S11, S12 and Table S4), Fe-BTC exhibited fast initial adsorption of Cr species, followed by a gradual desorption process that eventually reached equilibrium. We presume that the low removal efficiency of Fe-BTC for Cr(vi) at pH 3 (Fig. 2b) could be attributed to increased lability of the metal–ligand bond that could facilitate MOF destruction. However, Inductively Coupled Plasma Optical Emission Spectroscopy (ICP-OES) and Powder X-ray Diffraction (PXRD), carried out after Cr(vi) adsorption, do not indicate a significant decomposition of the MOF as only 2.20 ± 0.02% of the iron is leached (Fig. S13). Therefore, adsorption experiments were subsequently carried out at lower pH values of 2 and 1. Notably, there was complete degradation (Fig. S13) and loss of adsorption performance of the Fe-BTC at pH 1 (Fig. S14). We and others have previously shown that the introduction of hydrophobic coatings can improve the chemical stability of MOFs.^33–36^ Given the instability of Fe-BTC at pH 2 and below, we also attempted to coat the external surface of Fe-BTC/PS using two distinct coatings to prevent MOF degradation and subsequent polymer leaching all while preserving the Cr(vi) extraction capabilities. To construct the new materials, Fe-BTC/PS was coated with polydopamine (PDA), and then 1*H*,1*H*,2*H*,2*H*-perfluorodecanethiol (HSF) was grafted to the external surface *via* a Michael addition reaction (see the SI for details).^33^ The new composite, denoted Fe-BTC/PS@PDA-SF, retained its crystallinity and partial porosity with a BET surface area of 389 m^2^ g^−1^, which is much lower than that of Fe-BTC/PS, 1139 m^2^ g^−1^ (Fig. S15 and Table S1). As an alternative, Fe-BTC/PS was also coated with PDMS (polydimethylsiloxane) using previously published procedures.^37^ The composite, denoted as Fe-BTC/PS@PDMS was also crystalline and slightly more porous with a BET surface area of 467 m^2^ g^−1^ (Fig. S16 and Table S1).

**Fig. 3:**
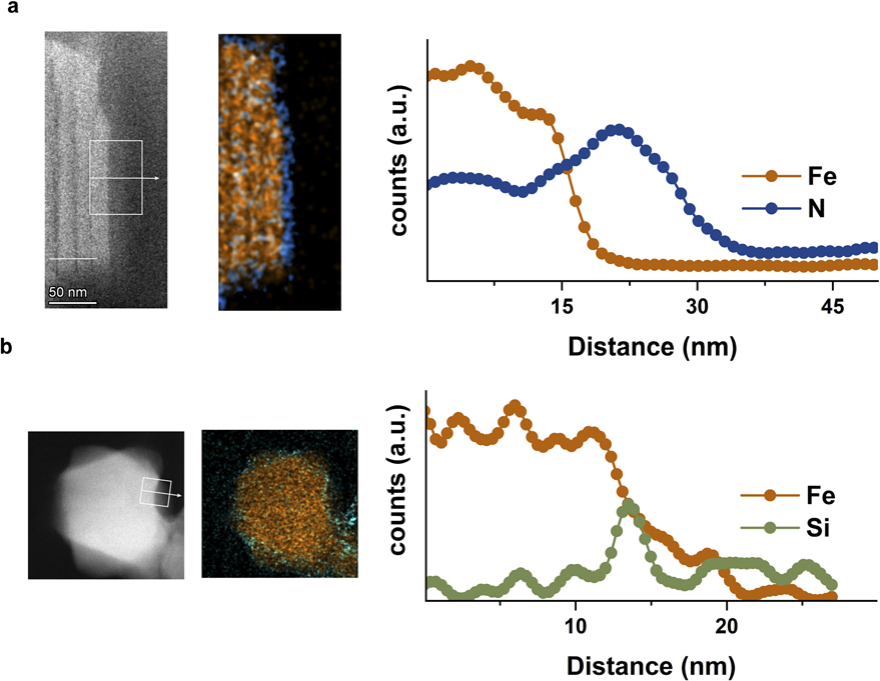
HAADF-STEM images and corresponding EDX elemental maps (left) and line scans (right) from a sliced crystallite of (a) Fe-BTC/PS@PDA-SF and (b) Fe-BTC/PS@PDMS.

To demonstrate that the hydrophobic coatings were located on the external surfaces of the crystallites rather than inside the MOF pores, STEM-EDX analysis was performed on approximately 60 nm-thick sections of Fe-BTC/PS@PDA-SF and Fe-BTC/PS@PDMS composites (Fig. 3a and b). Intensity line scans indicate that nitrogen counts extend beyond iron counts in Fe-BTC/PS@PDA-SF, confirming the PDA lies outside the MOF (Fig. 3a). Further, EDX data showed that fluorine, a signature of the appended HSF molecules, is only present on the external surface (Fig. S17). Next, XPS measurement of the F 1s region clearly shows signal associated with the new functional group added through a Michael addition, and TGA analysis reveals an increase in organics in the composite (Fig. S15). Similarly, the silicon line scan for Fe-BTC/PS@PDMS shows a signal extending beyond the iron counts, consistent with the PDMS being on the external surface of the Fe-BTC/PS particles (Fig. 3b). Last, contact angle measurements indicate values of 138.51° and 139.01° for Fe-BTC/PS@PDMS and Fe-BTC/PS@PDA-SF, respectively (Fig. S18 and Table S5), illustrating the highly hydrophobic nature of the external composite surfaces. To understand the performance of the materials, the kinetic parameters of the original composites and the coated ones were compared at pH = 3 (Fig. 2a). As expected, both coatings slowed the kinetics of initial Cr(vi) adsorption compared to Fe-BTC/PS (Fig. 2a, S11a and S12). This is reflected in the lower *k*_2_ values of 3.057 × 10^−4^ and 1.933 × 10^−4^ g mg^−1^ min^−1^ for Fe-BTC/PS@PDMS and Fe-BTC/PS@PDA-SF, respectively, compared to 1.8 × 10^−3^ g mg^−1^ min^−1^ for Fe-BTC/PS (Table S4). This likely stems from the decreased mass transfer within the coatings. Nevertheless, after 36 hours – both materials performed as well as or better than the non-coated material (Fig. S11). Further, batch adsorption experiments carried out at pH 2 indicate that the hydrophobic coatings do increase the stability of the material, which is reflected by the lower amount of Fe leached into solution (Fig. S13) and the higher Cr(vi) extraction efficiency of the two hydrophobic composites, when compared to Fe-BTC and the Fe-BTC/PS (Fig. 2b). Last, although the batch adsorption experiments at pH 1 also showed that the two hydrophobic composites remove significantly more Cr(vi) than Fe-BTC and the Fe-BTC/PS (Fig. S14), such conditions are too harsh, even for the coated materials, which is reflected in the leached iron into the aqueous solution (Fig. S13) and the materials' crystallinity loss (Fig. S19–S22).

As a second effort to make acid-stable MOF/polymer composites, *in situ* polymerization of PS was performed inside MOFs having reported acid stability including Zr-BDC and Cr-BDC (also known as UiO-66 and MIL-101, respectively) (Fig. S23 and S24). The selected materials were synthesized using a previously reported pH swing redox polymerization method^13^ (see SI). As a proof of concept, the resulting MOF/polymer composites were tested in acidic media at different pH (3, 2, and 1). As expected, all the composites outperformed the parent MOFs at pH 1–3 (Fig. S25 and S26). At pH 3, there was no Zr or Cr leaching from the composites as determined *via* ICP-OES. Further, PXRD measurements indicated that the materials remained crystalline (Fig. S27–S30). The same holds true for pH 2, where no metal leaching was observed. Unfortunately, as the pH was decreased to 1, the Zr-MOF began to undergo partial metal leaching; albeit, the quantity leached was low, ∼5% (Fig. S25 and S26). Cr-BDC appeared to be the most stable material, with Cr leaching below 0.1% (Fig. S26).

**Fig. 4:**
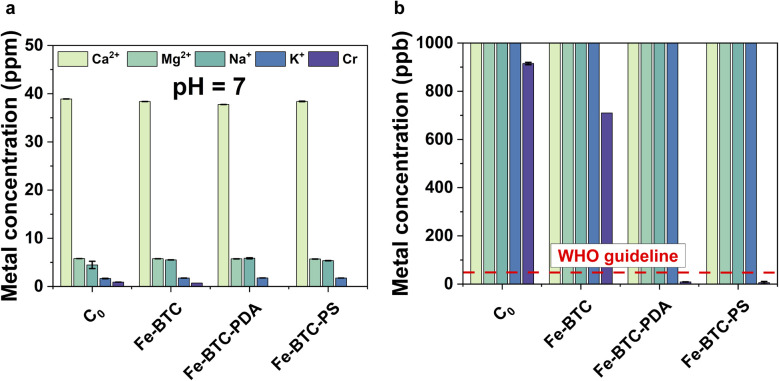
(a) Batch adsorption experiments in Cr(vi) spiked river water at pH 7, adsorption dosage of 0.5 g L^−1^, and an initial Cr concentration of 900 ppb; (b) zoomed in view of (a) showing low metal concentrations. The red dashed line indicates the WHO limit for chromium.

Last, Fe-BTC and the eight original Fe-BTC/polymer composites described above were also screened for the removal of toxic chromium, HCrO_4_^−^/CrO_4_^2−^, from natural water bodies (pH 7) (see SI, Fig. S31). From these tests, the two materials having the highest extraction efficiency from 1 and 10 ppm solutions are Fe-BTC/PDA and Fe-BTC/PS with capacities of 14.1 mg g^−1^ and 19.4 mg g^−1^, respectively (Fig. S31). Finally, these two composites were also tested in Rhone River water samples spiked with approximately 900 ppb of Cr(vi) as a competitive environment (Fig. 4). While both materials were able to reduce Cr(vi) in river water below the WHO recommended guideline (50 ppb^23^), Fe-BTC/PS showed superior removal performance when the adsorbent dosage was decreased to only 0.25 g L^−1^ for water treatment (Fig. S32). Additionally, within error of the experiment, interfering ions such as Ca^2+^, Mg^2+^, Na^+^ and K^+^ – were not removed from the river water, indicating the selective removal of Cr in complex water matrices.

## Conclusions

In this study, a MOF known as Fe-BTC was loaded with redox-active polymers, and the composites were tested for the removal of toxic Cr(vi) species, including HCrO_4_^−^ and HCrO_4_^−^/CrO_4_^2−^ from acidic (pH 1–3) and neutral aqueous streams (pH 7), respectively. While many of the MOF polymer composites were able to reduce the toxic Cr(vi) to the less toxic Cr(iii), the MOF functionalized with polyserotonin (PS) was found to be the best performer. While the composites function well, below pH 3 degradation was observed. To improve composite stability, two different approaches were explored, the use of hydrophobic coatings on Fe-BTC/PS and the use of other, more acid-stable MOFs as the porous support. While the coatings extended the utility to a pH 2, the use of more acid stable MOF, namely Cr-BDC, extended the utility to pH 1. The vision for this research is to strategically select MOF and polymer building blocks to design advanced composites able to extract valuable or toxic substances from aqueous media. It is hoped that such work can serve as a platform for the design of high-performance sorbents in the future.

## Author contributions

D. T. Sun, T. M. O. Felder, and W. L. Queen designed the work, T. M. O. Felder and W. Shi synthesized and characterized the materials. Data analysis was done by W. Shi and T. M. O. Felder. E. Oveisi performed electron microscopy characterizations. W. L. Queen coordinated the work. All authors discussed, read and commented on the manuscript.

## Conflicts of interest

There are no conflicts to declare.

## Supplementary Material

SC-OLF-D5SC05812K-s001

## Data Availability

Data for this article are available at Zenodo at https://doi.org/10.5281/zenodo.16599004. Supplementary information is available. See DOI: https://doi.org/10.1039/d5sc05812k.
