# Supplementary material for: Enhanced MOF performance in chromium(vi) removal from water using tailored MOF-polymer composites
Source: Chem Sci. 2025 Oct 14;16(45):21440–5. doi: 10.1039/d5sc05812k (PMC12531829; doi:10.1039/d5sc05812k)
Supplement: SC-016-D5SC05812K-s001 [file SC-016-D5SC05812K-s001.pdf]

Supplementary Information (SI) for Chemical Science.
This journal is © The Royal Society of Chemistry 2025

Enhanced MOF performance in chromium (VI) removal from water using tailored MOF-polymer composites

Timo M. O[a]. Felder, Wei Shi[b], Daniel T. Sun[a,d], Till Schertenleib[a], Emad Oveisi[c] Jordi Espín[a] and Wendy L. Queen*[a]

[a]Institute of Chemical Sciences and Engineering, École Polytechnique Fedérale de Lausanne (EPFL), CH-1950 Sion, Switzerland

[b]Research Center for Analytical Sciences, Department of Chemistry, College of Sciences, Northeastern University, Shenyang 110819, China

[c]Interdisciplinary Center for Electron Microscopy (CIME), École Polytechnique Fédérale de Lausanne (EPFL), CH-1015 Lausanne, Switzerland

[d]Sunchem Inc, 2300 4th St, Berkeley, CA 94710

[a] Timo M. O. Felder, Wei Shi, Till Schertenleib, Daniel T. Sun and Prof. W. L. Queen
Institute of Chemical Sciences and Engineering (ISIC), École Polytechnique, Fédérale de Lausanne, CH-1950, Sion , Switzerland
E-mail: wendy.queen@epfl.ch

[b] Wei Shi
Research Center for Analytical Sciences, Department of Chemistry, College of Sciences, Northeastern University, Shenyang 110819, China

[c] Emad Oveisi
Interdisciplinary Center for Electron Microscopy (CIME), École Polytechnique Fédérale de Lausanne (EPFL), CH-1015 Lausanne, Switzerland

[d] Daniel T. Sun
Sunchem Inc, 2300 4th St, Berkeley, CA 94710

Table of Contents

Synthesis of MOFs.....5

Fe-BTC (MIL-100(Fe)).....5

Zr-BDC (UiO66).....5

Zr-BDPC (UiO67).....5

Cr-BDC (MIL-101(Cr)).....5

Synthesis of MOF/polymer Composites.....6

Fe-BTC/PS (serotonin).....6

Fe-BTC/PoAP (poly -ortho-aminophenol).....6

Fe-BTC/PmAP (meta-aminophenol).....7

Fe-BTC/PpAP (para-aminophenol).....7

Fe-BTC/PoPDA (o-phenylenediamine).....7

Fe-BTC/mPDA (m-phenylenediamine).....7

Fe-BTC/pPDA (p-phenylenediamine).....8

Fe-BTC/PDA (dopamine).....8

Zr-BDC/PS.....8

Cr-BDC-PS.....8

Synthesis of coated MOF-polymer composite.....9

Fe-BTC/PS@PDA-SF.....9

Fe-BTC/PS@PDMS.....9

Methods Characterization.....10

Powder X-ray Diffraction.....10

N_2 Adsorption Isotherms.....10

Thermogravimetric analysis (TGA).....10

X-ray Photoelectron Spectroscopy.....10

Electron Microscopy – Electron Dispersive X-ray (EDX) Spectroscopy.....10

UV-VIS Adsorption Measurements.....11

Methods chromium extraction.....12

Heavy metal experiments at acidic and neutral pH (1, 2, 3 and 7).....12

Kinetic experiments (pH 3).....12

Cr(VI) adsorption isotherm (pH 3).....12

Cr(VI) spiked river water adsorption experiments.....12

Calculation adsorption parameter.....13

Characterization ... 15
Characterization Fe-based MOF/polymer ... 15
Fe-BTC/PS (polyserotonine) ... 15
Fe-BTC/PoAP / PmAP / PpAP (poly - ortho, meta, para-aminophenol) ... 16
Fe-BTC/ PoPDA / mPDA / PpPDA (poly - ortho, meta, para – phenylenediamine) ... 17
Fe-BTC/PDA (dopamine) ... 18
BET analysis of MOF/polymer composites ... 19
Overview polymer loading for al MOF/polymer composites ... 20
Selection of best performing material in acidic conditions ... 21
Optimal polymer loading ... 21
XPS analysis of N 1S region of Fe-BTC/PS ... 22
Adsorption isotherm pH = 3 ... 23
Cr(III) adsorption experiment ... 24
Kinetic performance of the materials at pH = 3 ... 24
Fitted adsorption experiment using pseudo second order model ... 25
Stability of Fe-BTC, Fe-BTC/PS and coated hydrophobic materials in acidic conditions ... 26
Performance of Fe-BTC, Fe-BTC/PS and coated hydrophobic materials in acidic conditions ... 27
Characterization coated MOF/polymer composites ... 28
Fe-BTC/PS-@PDA-SF ... 28
Fe-BTC/PS@PDMS ... 29
STEM-EDX analysis of coated materials Fe-BTC/PS@PDA-SF ... 30
Contact angle Fe-BTC/PS@PDMS and Fe-BTC/PS@PDA-SF ... 30
PXRD analysis of Fe-BTC, Fe-BTC/PS, and coated materials in acidic conditions ... 31
Characterization different metal-based MOF/polymer ... 34
Zr-BDC/PS ... 34
Cr-BDC-PS ... 35
Performance of other MOF-PS composites in acidic conditions ... 36
Stability of other MOF-PS composites in acidic conditions (pH 3) ... 37
Selection of best performing material in neutral (pH=7) conditions ... 39
Performance of Fe-BTC/PDA and Fe-BTC/PS in real river water conditions ... 40
References ... 41

Synthesis of MOFs

Fe-BTC (MIL-100(Fe))

9.72 g of $FeCl_3 \cdot 6H_2O$, 3.36 g of trimesic acid and 120 mL of distilled water were added to a 180 mL autoclave and sealed. The reaction vessel was put inside an oven and heated to 130 °C for 72 hours. After the reaction cooled to room temperature the orange slurry was centrifuged at 7000 rpm and was washed with copious amounts of methanol and then dried under vacuum. The resulting powder was loaded into a double thickness whatman cellulose thimble and underwent soxhlet purification with methanol for 24 hours. After purification the final product was dried under vacuum.

Zr-BDC (UiO66)

Zr-BDC was synthesized according to previous reported literature procedure.[1] In short, 2.41 g of $ZrCl_4$ was dissolved in DMF (180 mL) in a 1L cap-screw vessel and 18 mL of HCl (37 wt. %) was added. Then, 2.22g of Terephthalic acid was separately dissolved in DMF (360 mL), sonicated and added to the mixture. The final reaction mixture was additionally sonicated for 15 minutes followed by heating at 80°C for 24 hours. After the reaction was allowed to cool to r.t., the crude was placed in 50 mL centrifugation vessels, washed with DMF (3 x 40 mL DMF) and solvent exchanged with EtOH (3 x 40 mL, overnight). The final product was dried at r.t. under vacuum for 12 hours, yielding Zr-BDC as a white crystalline solid.

Cr-BDC (MIL-101(Cr))

In a 1 L Teflon jar 52.8 g $Cr(NO_3)_3 \cdot 9H_2O$ (0.132 mol), 18.0 g BDC (0.108 mol) were dispersed in 660 mL deionized H_2O and 8.56 mL HNO_3 69% (0.132 mol, 1 eq respect chromium nitrate) were added. The mixture was stirred for 30 min, and then placed in an autoclave to a preheated oven at 200 °C for 16 h. The reaction mixture was allowed to cool down naturally in the oven for 12 h. Next, the supernatant was decanted to collect the solid in six 50 mL falcon tubes by centrifugation for 10 min at 7800 rpm. Again, the supernatant was discarded and the sample was washed once with 35 mL DMF/falcon tube shaking them for 10 min. The solid was then transferred to a 500 mL glass jar, where 200 mL of fresh DMF were added. After stirring at 700 rpm for 6 h, the solids were centrifuged, the supernatant was discarded. This process was repeated for 3 more times to ensure proper removal of unreacted species. The same washing procedure was done for ethanol. After that, dry in the vacuum oven at room temperature for 24 h.

Synthesis of MOF/polymer Composites

Fe-BTC/PS (serotonin)

Fe-BTC/PS (PS – polyserotonin) was synthesized using the double solvent method that we previously reported.[2] First, in a 2-neck 500 mL round bottom flask, 1 g of Fe-BTC was activated at 150 °C under vacuum using a schlenk line and a rough oil pump overnight. After activation the reaction vessel was flushed with nitrogen and allowed to cool down to room temperature. Next,

80 mL of hexane was added and the reaction mixture was stirred for 1 hour. In a separate vial, 1.67 g of serotonin HCl was dissolved in 2 mL of distilled water and then subsequently added to the reaction mixture. In less than an hour the solids settled down with the water to the bottom of the 2-neck round bottom flask. The hexane was decanted and then 1.6 mL of 25% NH_3 in 50 mL of ethanol was added to the reaction. Afterwards the black mixture was stirred overnight at ambient conditions. After reaction completion, the solids were separated using a centrifuge at 7000 rpm. The black powder was purified using soxhlet extraction with ethanol overnight and the final product was dried under vacuum.

Fe-BTC/PoAP (poly -ortho-aminophenol)

First, 0.250 Fe-BTC was activated in a 250 mL two-neck round bottom flask under vacuum at 150 °C overnight. After activation the reaction vessel was cooled down to room temperature and flushed with nitrogen. In a dry box, 0.125 g of 2-aminophenol (oAP) was added with 50 mL anhydrous methanol in a separate flask. Using a steel cannula and N_2, the ethanol solution was transferred to the flask containing the activated Fe-BTC. The reaction mixture was stirred for 1 hour. Subsequently, the reaction mixture was then exposed to air and heated to 50 °C for 24 hours. After reaction completion, the solids were separated using a centrifuge at 7000 rpm. The black powder was purified using soxhlet extraction with ethanol overnight and the final product was dried under vacuum.

Fe-BTC/PmAP (meta-aminophenol)

First, 0.250 Fe-BTC was activated in a 250 mL two-neck round bottom flask under vacuum at 150 °C overnight. After activation the reaction vessel was cooled down to room temperature and flushed with nitrogen. In a dry box, 0.129 g of 3-aminophenol (mAP) was added with 50 mL anhydrous ethanol in a separate flask. Using a steel cannula and N_2, the ethanol solution was transferred to the flask containing the activated Fe-BTC. The reaction mixture was stirred for 1 hour. Subsequently, the reaction mixture was then exposed to air and heated to 50 °C for 24 hours. After reaction completion, the solids were separated using a centrifuge at 7000 rpm. The powder was purified using soxhlet extraction with ethanol overnight and the final product was dried under vacuum.

Fe-BTC/PpAP (para-aminophenol)

First, 0.250 Fe-BTC was activated in a 250 mL two-neck round bottom flask under vacuum at 150 °C overnight. After activation the reaction vessel was cooled down to room temperature and flushed with nitrogen. In a dry box, 0.129 g of 4-aminophenol (pAP) was added with 50 mL anhydrous ethanol in a separate flask. Using a steel cannula and N_2, the ethanol solution was transferred to the flask containing the activated Fe-BTC. The reaction mixture was stirred for 1 hour. Subsequently, the reaction mixture was then exposed to air and heated to 50 °C for 24 hours. After reaction completion, the solids were separated using a centrifuge at 7000 rpm. The black powder was purified using soxhlet extraction with ethanol overnight and the final product was dried under vacuum.

Fe-BTC/PoPDA (o-phenylenediamine)

First, 0.250 g Fe-BTC was activated at 150 °C under vacuum using a two-neck round bottom flask, a schlenk line and a rough oil pump overnight. After activation, the reaction vessel was cooled down to room temperature and nitrogen was introduced into the system. 0.125 g of o-phenylenediamine (2-phenylenediamine) and 50 mL of methanol were mixed together in a dry box. The air free methanol solution was transferred to the reaction vessel with the activated MOF via a steel cannula and allowed to stir at 250 rpm for 1 hour. The reaction vessel was then exposed to air and heated to 50 °C for 24 hours. After reaction completion, the powder was separated by centrifugation. The powder was purified using soxhlet extraction with ethanol overnight and the final product was dried under vacuum.

Fe-BTC/mPDA (m-phenylenediamine)

First, 0.250 g Fe-BTC was activated at 150 °C under vacuum using a two-neck round bottom flask, a schlenk line and a rough oil pump overnight. After activation, the reaction vessel was cooled down to room temperature and nitrogen was introduced into the system. 0.125 g of m-phenylenediamine (3-phenylenediamine) and 50 mL of methanol were mixed together in a dry box. The air free methanol solution was transferred to the reaction vessel with the activated MOF via a steel cannula and allowed to stir at 250 rpm for 1 hour. The reaction vessel was then exposed to air and heated to 50 °C for 24 hours. After reaction completion, the powder was separated by

centrifugation. The powder was purified using soxhlet extraction with ethanol overnight and the final product was dried under vacuum.

Fe-BTC/pPDA (p-phenylenediamine)

First, 0.25 g Fe-BTC was activated at 150 °C under vacuum using a two-neck round bottom flask, a schlenk line and a rough oil pump overnight. After activation, the reaction vessel was cooled down to room temperature and nitrogen was introduced into the system. 0.125 g of p-phenylenediamine (4-phenylenediamine) and 50 mL of methanol were mixed together in a dry box. The air free methanol solution was transferred to the reaction vessel with the activated MOF via a steel cannula and allowed to stir at 250 rpm for 1 hour. The reaction vessel was then exposed to air and heated to 50 °C for 24 hours. After reaction completion, the powder was separated by centrifugation. The powder was purified using soxhlet extraction with ethanol overnight and the final product was dried under vacuum.

Fe-BTC/PDA (dopamine)

First, 0.250 Fe-BTC was activated in a 250 mL two-neck round bottom flask under vacuum at 150° C overnight. After activation the reaction vessel was cooled down to room temperature and flushed with nitrogen. In a dry box, 0.25 g of free base dopamine was added with 50 mL anhydrous ethanol in a separate flask. Using a steel cannula and N2, the ethanol solution was transferred to the flask containing the activated Fe-BTC. The reaction mixture was stirred for 1 hour. Subsequently, the reaction mixture was then exposed to air and stirred for 24 hours. After reaction completion, the solids were separated using a centrifuge at 7000 rpm. The black powder was purified using Soxhlet extraction with ethanol overnight and the final product was dried under vacuum.

Zr-BDC/PS

In a 2-neck 500 mL round bottom flask 238 mg of Zr-BDC was activated at 150 °C under vacuum using a Schlenk line technique. After activation the reaction vessel was flushed with nitrogen and allowed to cool down to room temperature. Next, 20 mL of hexane was added, and the reaction mixture was stirred for 1 hour. In a separate flask, 0.40 g of Serotonin HCl was dissolved in 0.5 mL of distilled water and added to the round bottom flask. After 1 hour the solids settled down to the bottom of the round bottom flask. Next, the hexane was decanted and then 0.8 mL of 25% NH_3 in 12.5 mL of ethanol was added to the reaction. Afterwards the solid turned black and was additionally stirred overnight at ambient conditions. Next, the solids were separated using a centrifuge at 7000 rpm. The black powder was purified using Soxhlet extraction with ethanol overnight and the final product was dried under vacuum.

Cr-BDC-PS

In a 2-neck 500 mL round bottom flask 238mg of Cr-BDC was activated at 150 °C under vacuum using a Schlenk line technique. After activation the reaction vessel was flushed with nitrogen and allowed to cool down to room temperature. Next, 20 mL of hexane was added, and the reaction mixture was stirred for 1 hour. In a separate flask, 0.40 g of serotonin HCl was dissolved in 0.5 mL

of distilled water and added to the round bottom flask. After 1 hour the solids settled down to the bottom of the round bottom flask. Next, the hexane was decanted and then 0.4 mL of 25% NH_3 in 12.5 mL of ethanol was added to the reaction. Then, the reaction was additionally stirred overnight while a constant flow of compressed air was flown through the reaction mixture using a needle. Next, the solids were separated using a centrifuge at 7000 rpm. The black powder was purified using Soxhlet extraction with ethanol overnight and the final product was dried under vacuum.

Synthesis of coated MOF-polymer composite

Fe-BTC/PS@PDA-SF

Fe-BTC/PS@PDA-SF (PDA-SF – polydopamine-perfluorodecanethiol) was synthesized using methods we previously reported.[3] First, 0.500 g of Fe-BTC/PS was sonicated in 200 mL of methanol for 10 minutes. After, 0.500 g of free base dopamine was added to the reaction mixture and was stirred overnight under ambient conditions. The solids were then separated using a centrifuge at 7000 rpm and subsequently washed with fresh methanol three times. Afterwards the resulting powder was sonicated in 200 mL of methanol for ten minutes. 0.595 mL of 1H,1H,2H,2H-Perfluorodecanethiol and 0.595 mL of triethylamine were then added to the mixture and stirred overnight under ambient conditions. The resulting powder was separated using a centrifuge at 7000 rpm and washed with toluene followed by methanol (3 x 40 mL). Fe-BTC/PS@PDA-PF was dried by vacuum overnight and activated at 125 °C before standard characterization.

Fe-BTC/PS@PDMS

Fe-BTC/PS@PDMS was synthesized adapting literature procedure.[4] First, 180 mg PDMS dissolved in 30 ml hexane. To this 0.3 g Fe-BTC/PS were added and sonicated for 10 min. Subsequently, the sample was filtered and put in an oven at 70 °C for 30 min. After drying at 70°C, the powder was additionally dried by vacuum overnight and was activated at 125 °C before standard characterization.

Methods Characterization

Powder X-ray Diffraction

Powder X-ray diffraction was performed on a Bruker D8 Discover system. The lab source is for the instrument is a Cu Kα source (1.54056 Å) at 40 kV and 40 mA. The primary optics slit and secondary optics slit were set to 12 mm and 9 mm respectively with a NiO filter. The powder samples were ground with a mortar and pestle and then loaded onto a 1 mm deep sample holder or a zero-background holder. Simulated powder patterns were generated using Vesta crystallography software. For samples measured after chromium adsorption experiments at different pH, the measurement was carried out in transmission mode.

N_2 Adsorption Isotherms

All N_2 adsorption measurements were performed on a Belsorp Max II instrument. Before measurements 50-100 mg of sample was activated at 125 °C under vacuum overnight in glass sample cells. The samples were then cooled to room temperature, backfilled with argon, and transferred to the Belsorp Max II adsorption analyzer. Nitrogen adsorption isotherms were collected at 77 K. After the isotherms were generated the surface areas (m^2/g) were calculated using the BETSI software.[5]

Thermogravimetric analysis (TGA)

The thermogravimetric analysis curve was obtained using a TA Q-Series TGA Q500. Samples were loaded onto a tared platinum pan. The balance flow rate was at 40 mL/min with nitrogen and the sample flow rate was at 40 mL/min with air heating at a rate of 5 °C per minute.

X-ray Photoelectron Spectroscopy

X-ray photoelectron spectroscopy measurements were carried out using Kratos Axis Supra Instrument with monochromated Al Kα X-ray source. The data was analyzed using the CasaXPS software.

Electron Microscopy – Electron Dispersive X-ray (EDX) Spectroscopy

Scanning transmission electron microscopy (STEM) and energy-dispersive X-ray spectroscopy (EDX) were performed on a double Cs-corrected Thermo Fisher Scientific Titan-Themis 60-300 operated in scanning mode at an accelerating voltage of 200 kV. This microscope is equipped with a high-brightness field emission gun (X-FEG), a Super-X EDS system comprising four silicon drift detectors, and Velox acquisition software. STEM images were acquired in high-angle annular dark-field (HAADF) configuration. STEM-EDX elemental maps were collected as spectrum images, in which a focused electron probe was scanned in a raster pattern across a region of interest. Sample preparation for STEM measurements was carried out using ultramicrotomy. A resin-embedded composite was serially sectioned into approximately 60 nm-thick slices, which were then deposited onto a TEM grid with an ultrathin carbon support film.

UV-VIS Adsorption Measurements

For the detection of Cr(VI) in solution the solution were measured via UV-VIS method.[6] In an acidic media, Cr(VI) reacts with diphenylcarbazide to form a purple compound, which can be measured spectrophotometrically at a wavelength of 540 nm. For the calibration curve, a 100 ppm stock solution of Cr(VI) was prepared by dissolving 282.9 mg of potassium dichromate ($K_2Cr_2O_7$) in Milli-Q water (1L). From this stock solution, different concentrations of Cr(VI) solution were prepared. Then, 15 µL of diluted sulfuric acid solution (Volume 1 : 1 / sulfuric acid : water) and 15 µL of diluted phosphoric acid solution (Volume 1 : 1 / phosphoric acid : water) were added to 1.5 mL of Cr(VI) solution (0.05 ppm, 0.08 ppm, 0.1 ppm, 0.2 ppm, 0.5 ppm, 0.8 ppm and 1ppm). Next, 60 µL of a solution of 1,5-diphenylcarbazide (DPC) in acetone (200 mg in 100mL) was added. After approx. 5 min, the absorption spectrum between 640-440nm was measured. To measure the Cr(VI) content after adsorption experiment, the samples were filtered using a 25 mm hydrophilic PTFE membrane syringe filter with 0.22 mm pore to remove and solids. Then, the samples were analyzed the same way as the standard solution.

Polymer loading calculation

Polymer loadings were calculated from TGA data using following equation;

$$polymerloading\ (wt.\%) = 1 - \frac{residual\ weight\ MOF/polymer}{residual\ weight\ bare\ MOF}$$

Methods chromium extraction

Metal concentrations in aqueous solutions were determined using an Agilent 5110 synchronous vertical dual view ICP-OES or a Perkin-Elmer NexIon 350 D ICP-MS for ultra-low concentrations. All samples were acidified to a 2% HNO_3 before analysis.

Heavy metal experiments at acidic and neutral pH (1, 2, 3 and 7)

In general, 5 mg of material was added to 20 mL Milli-Q water spiked with Cr(VI) (K_2CrO_4) (either 10 ppm or 20 ppm). The pH was adjusted to the desired level of 1, 2, 3 and 7 using solution of 0.01M HCl and 0.01M NaOH. Next, the samples were placed in a Thermo Scientific MaxQ4450 Orbital Shaker for 24 hours at 200 rpm at r.t.. Afterward, the water samples were filtered using a 25 mm hydrophilic PTFE membrane syringe filter with 0.22 μm pore to remove the adsorbent. The water solutions were acidified to a 2% nitric acid solution and elemental analysis was carried out.

Kinetic experiments (pH 3)

For kinetic experiment, 30 mg of material were added to a 20 ppm Cr(VI) solution (120 mL). Aliquots (1mL) samples were taken at different time points and filtered using a 25 mm hydrophilic PTFE membrane syringe filter with 0.22 μm pore to remove the adsorbent. The water solutions were acidified to a 2% nitric acid solution and measured by ICP-OES.

Cr(VI) adsorption isotherm (pH 3)

For adsorption isotherm, 5 mg of samples were added to a solution with different concentration of Cr(VI) (1-100ppm). After 24 hours, the water samples were filtered using a 25 mm hydrophilic PTFE membrane syringe filter with 0.22 μm pore to remove the adsorbent. The water solutions were acidified to a 2% nitric acid solution and elemental analysis was carried out.

Cr(VI) spiked river water adsorption experiments

River water taken from the Rhone river in Sion (Sion, Switzerland, Latitude: 46.228332, Longitude: 7.369975) was spiked with 900 ppb of Cr(VI) (K_2CrO_4). Approximately 5 mg of MOF/polymer composite was added to a 10, 20 and 30 mL solution of simulated hexavalent chromium contaminated water and placed in a Thermo Scientific MaxQ4450 Orbital Shaker for 24 hours at 200 rpm at r.t.. The pH was adjusted to 7 using 0.01M HCl and 0.01M NaOH solutions. Afterward, the water samples were filtered using a 25 mm hydrophilic PTFE membrane syringe filter with 0.22 μm pore to remove the adsorbent. The water solutions were acidified to a 2% nitric acid solution and elemental analysis was carried out.

Calculation adsorption parameter

The adsorption capacity Q_e - mg removed metals per gram of adsorbent at equilibrium - of the materials was calculated using the following equation:

$$Q_e = \frac{(C_o - C_e)V}{m}$$

Where, C_o is the initial concentration, C_e is the end concentration, V is the volume and m is the mass of the composite.

The removal rate was calculated using the following equation:

$$removal\ percentage\ (\%) = \frac{C_o - C_e}{C_o} x100$$

Where, C_o is the initial concentration, C_e is the end concentration

For kinetic experiments, the data was fitted using PSO model (Pseudo second order model). The PSO equation is given below:

$$\frac{t}{q_t} = \frac{1}{k^2 {q_e}^2} + \frac{t}{q_e}$$

q_e = the amount of chromium adsorbed at equilibrium (mg/g)

k = rate constant for PSO model (g/ (mg min))

t = time (min)

The chromium adsorption isotherm was fitted using two different equations. The Langmuir and the Freundlich equation are given below:

$$Q_e = \frac{q_m b C_e}{1 + (bC_e)}$$

Q_e = the amount of chromium adsorbed at equilibrium (mg g^{-1})

C_e = the concentration of chromium at equilibrium (mg L^{-1})

q_m = the maximum adsorption capacity (mg g^{-1})

b = Langmuir constant (L mg^{-1})

$$Q_e = K_F C_e^{1/n}$$

Q_e = the amount of chromium adsorbed at equilibrium (mg g^{-1})

C_e = the concentration of chromium at equilibrium (mg L^{-1})

K_F = the affinity constant for adsorption

n = the index of heterogeneity

Characterization

Characterization Fe-based MOF/polymer

Fe-BTC/PS (polyserotonine)

PXRD, TGA, XPS and N_2 adsorption isotherm for of Fe-BTC/PS is presented in **Figure S1**. Polymer loading calculated from TGA can be found in **Table S2**.

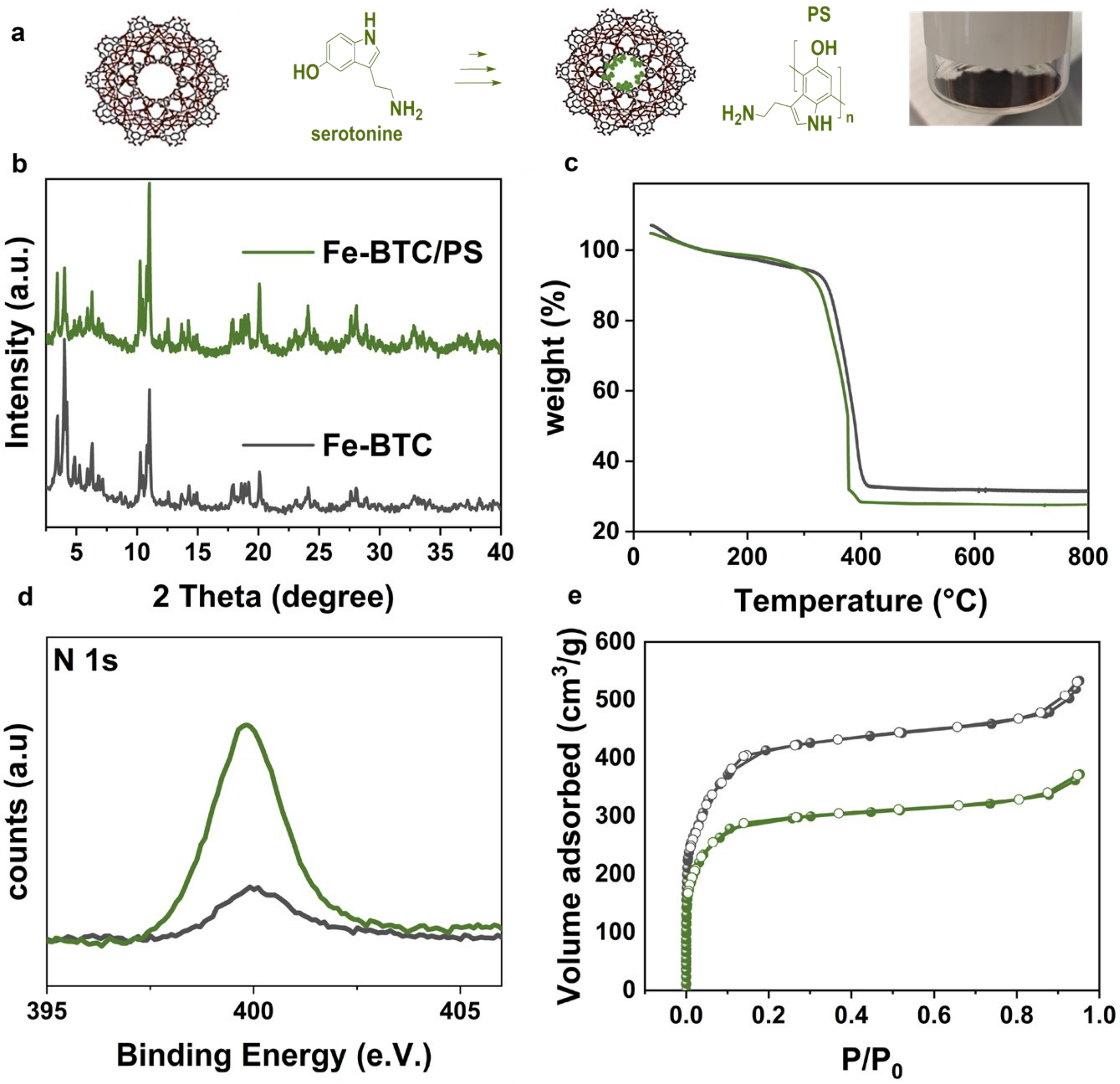

Figure S 1 Characterization of Fe-BTC/PS: a) Schematic representation of reaction to Fe-BTC/PS including a photograph of obtained powder. b) Powder X-ray diffraction patterns of the Fe-BTC (black) and Fe-BTC/PS (green). c) Thermogravimetric analysis curves. d) XPS N 1S region. e) N_2 adsorption isotherms.

Fe-BTC/PoAP / PmAP / PpAP (poly - ortho, meta, para-aminophenol)

PXRD, TGA, XPS and N_2 adsorption isotherm for polymers synthesized using ortho, meta and para-aminophenol (oAP, mAP and pAP) are presented in **Figure S2**. Polymer loadings calculated from TGA can be found in **Table S2**.

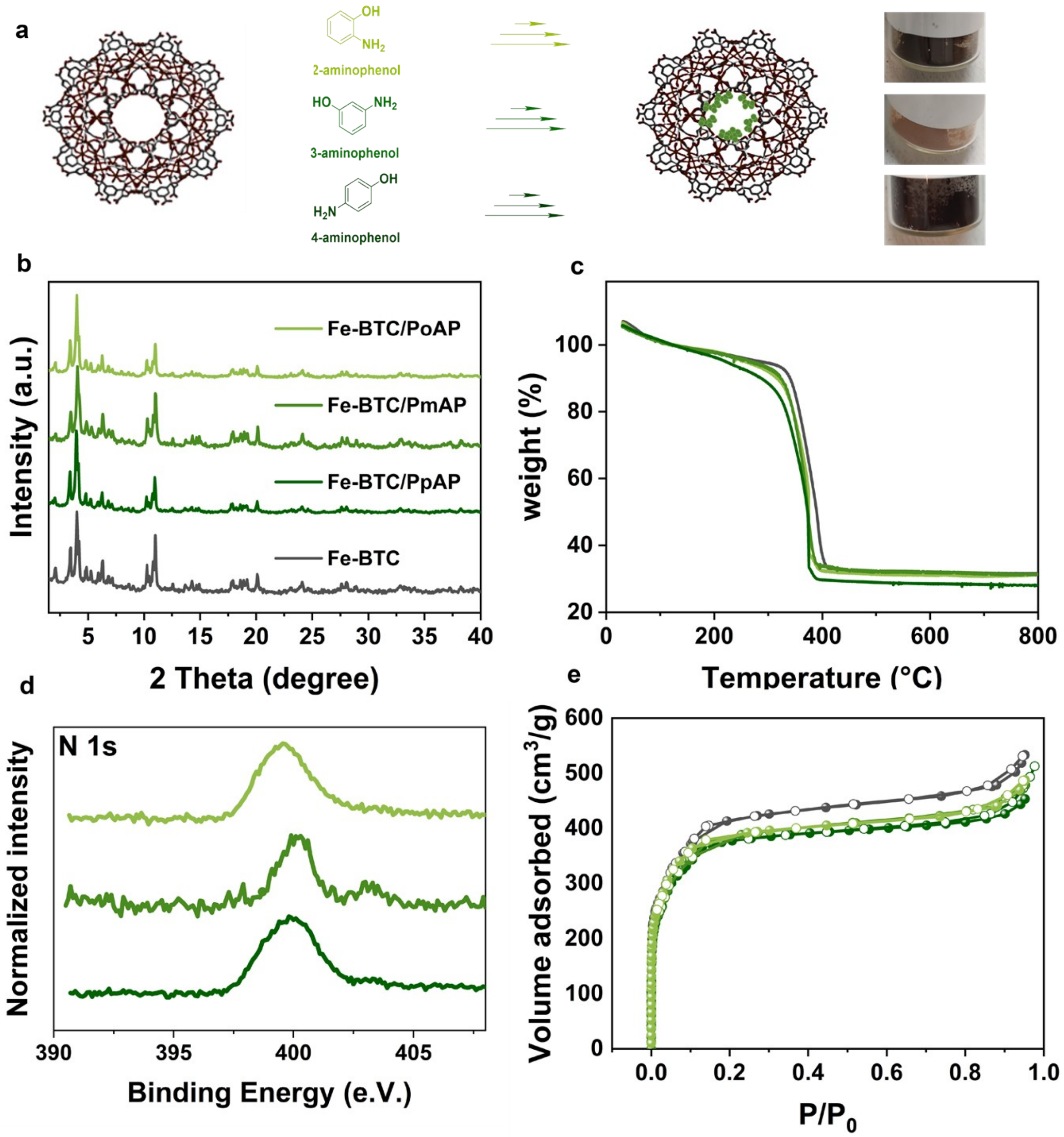

Figure S 2 Characterization incorporated aminophenol polymer in Fe-BTC (grey) – from light to dark green: Fe-BTC-PoAP / PmAP / PpAP. a) Schematic representation of reaction to Fe-BTC with monomers including a photograph of the obtained powders. b) Powder X-ray Diffraction patterns. c) Thermogravimetric analysis curves. d) Normalized XPS N 1S region. e) N_2 adsorption isotherms.

Fe-BTC/ PoPDA / mPDA / PpPDA (poly - ortho, meta, para – phenylenediamine)

PXRD, TGA, XPS and N_2 adsorption isotherm for polymers synthesized using ortho, meta and para-phenylenediamine (oPDA, mPDA and pPDA) are presented in **Figure S3**. Polymer loadings calculated from TGA can be found in **Table S2**. For mPDA, did not observe any difference in the TGA compared to Fe-BTC, therefore we did not calculate any polymer loading.

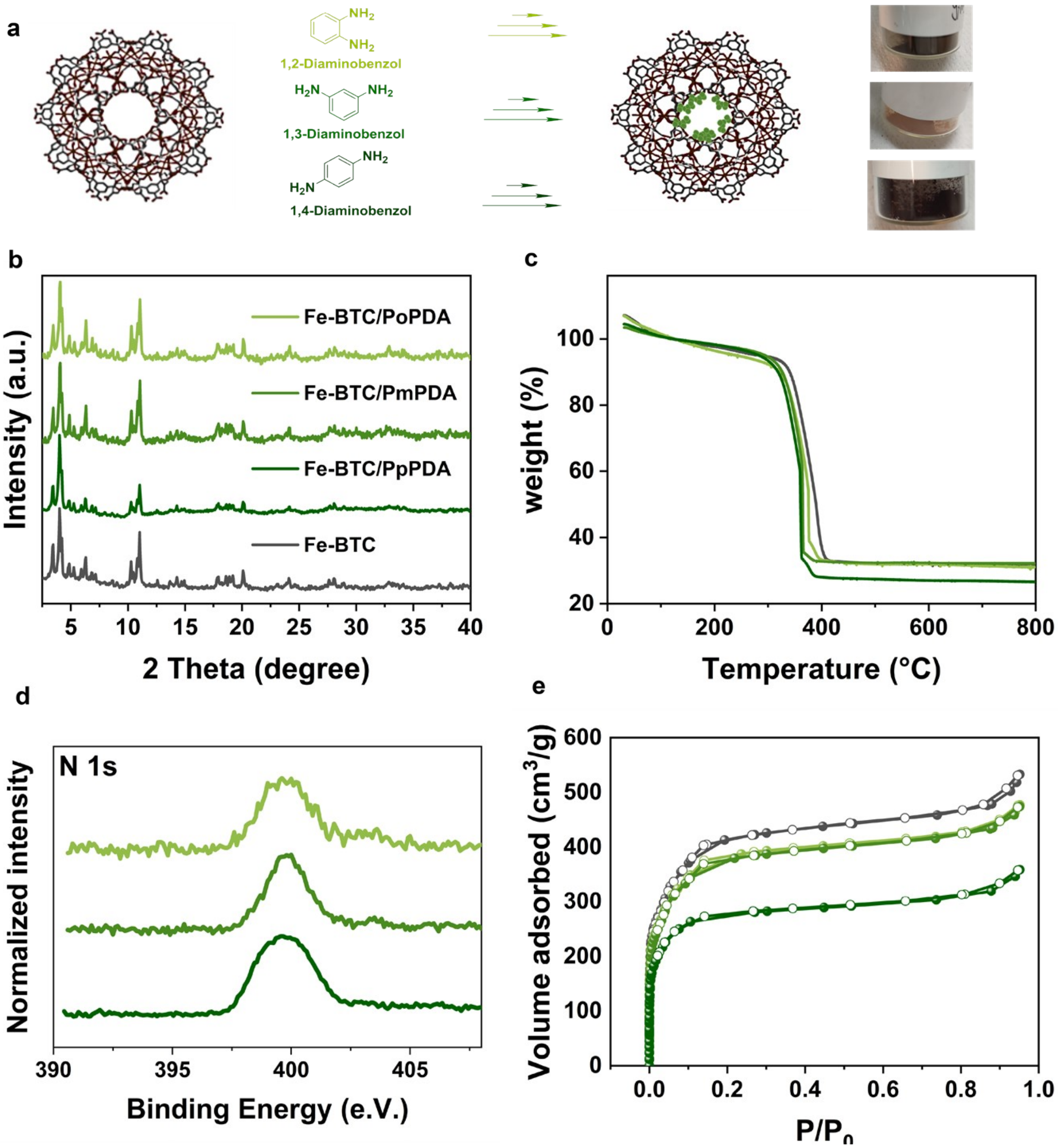

Figure S 3 Characterization incorporated phenylenediamine polymer in Fe-BTC (grey) – from light to dark green Fe-BTC / PoPDA / PmPDA / PpPDA. a) Schematic representation of reaction to Fe-BTC with monomers including a photograph of the obtained powders. b) Powder X-ray diffraction patterns. c) Thermogravimetric analysis curves. d) Normalized XPS N 1S region. e) N_2 adsorption isotherms.

Fe-BTC/PDA (dopamine)

PXRD, TGA, XPS and N_2 adsorption isotherm for Fe-BTC/PDA is presented in **Figure S4**. Polymer loading calculated from TGA can be found in **Table S2**.

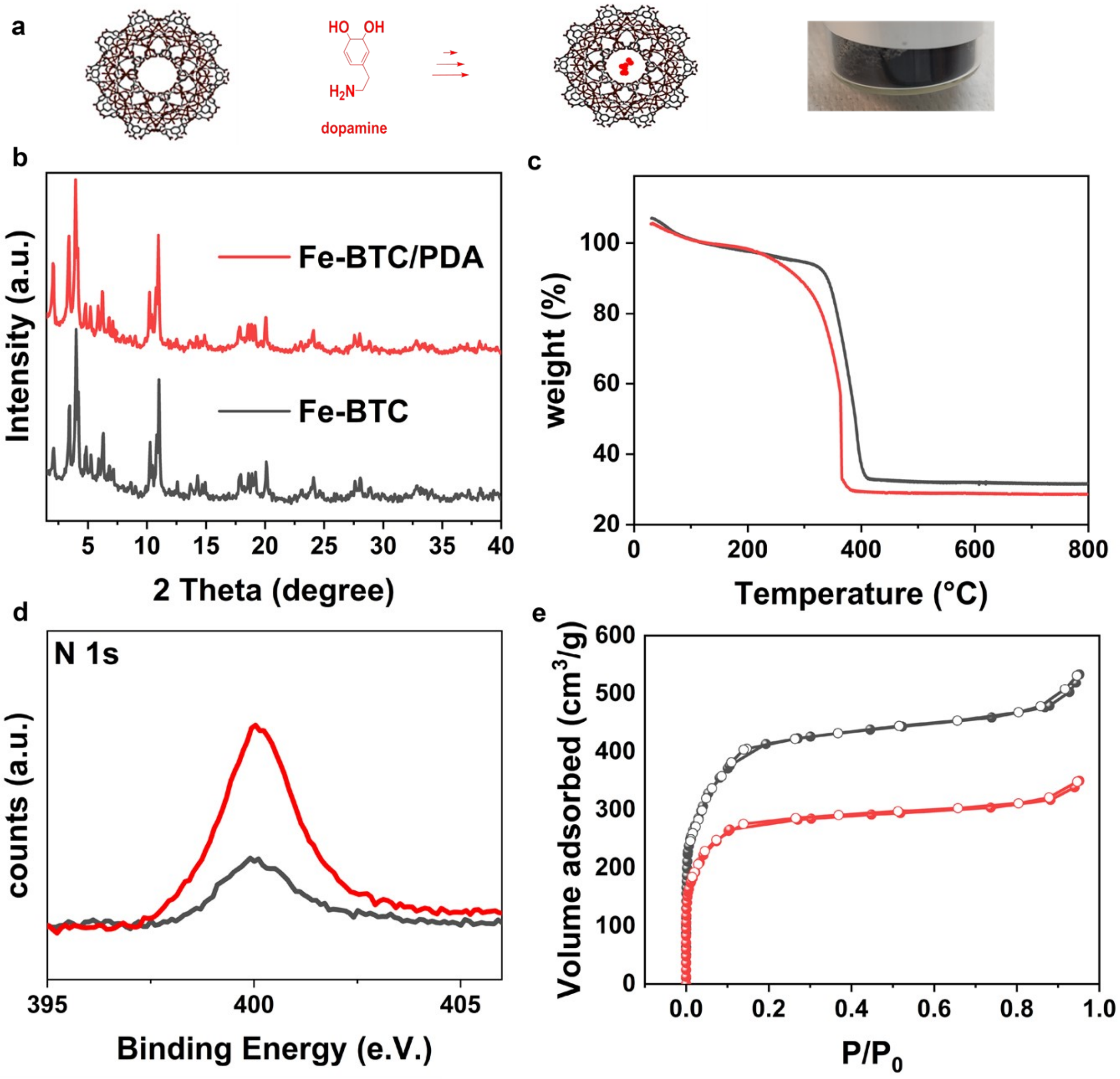

Figure S 4 Characterization of Fe-BTC/PDA: a) Schematic representation of reaction to Fe-BTC/PDA including a photograph of obtained powder. b) Powder X-ray diffraction patterns of the Fe-BTC (black) and Fe-BTC/PDA (red). c) Thermogravimetric analysis curves. d) XPS N 1S region Fe-BTC/PDA. e) N_2 adsorption isotherm.

BET analysis of MOF/polymer composites

Table S 1 Overview of calculated Brunauer–Emmett–Teller (BET) surface areas for all materials obtained using BETSI[5]

Material	BET (m^2/g)
Fe-BTC	1556
Fe-BTC/PS	1139
Fe-BTC/PS@PDMS	467
Fe-BTC/PS@PDA-SF	389
Fe-BTC/PDA	1050
Fe-BTC/PoPDA	1430
Fe BTC/PmPDA	1408
Fe-BTC/PpPDA	1068
Fe-BTC/PoAP	1511
Fe-BTC/PmAP	1439
Fe-BTC/PpAP	1505
Zr-BDC	1616
Zr-BDC/PS	763
Cr-BDC	3247
Cr-BDC/PS	1705

Overview polymer loading for al MOF/polymer composites

Table S 2 MOF polymer loading determined by TGA normalized at 125°C unless otherwise stated.

Material	polymer loading wt. % calculated by TGA
Fe-BTC/PS	12.8
Fe-BTC/PDA	11.3
Fe-BTC/PoPDA	2.3
Fe-BTC/PmPDA	-$^{\alpha}$
Fe-BTC/PpPDA	16
Fe-BTC/PoAP	2.1
Fe-BTC/PmAP	1.5
Fe-BTC/PpAP	11.1
Zr-BDC/PS	7.3$^{\beta}$
Cr-BDC/PS	26

$^{\alpha}$ *No difference in TGA after polymer incorporation*

$^{\beta}$ *TGA data was normalized at 300°C which marked the beginning of weight loss for Zr-BDC-PS.*

Selection of best performing material in acidic conditions

Complementary to **Figure 1**, **Figure S5** shows the adsorption capacity (Q_e) for the removal of Cr(VI) for different MOF/polymer composites at pH 3.

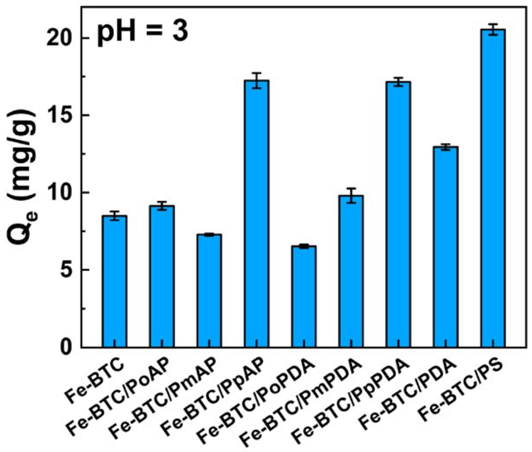

Figure S 5 Overview results batch adsorption experiment in acidic pH (pH=3) analyzed by ICP-OES for all MOF/polymer composites. Conditions: Adsorption dosage = 0.25g/L, adsorption time = 24 hours and initial Cr(VI) concentration of 10 ppm.

Optimal polymer loading

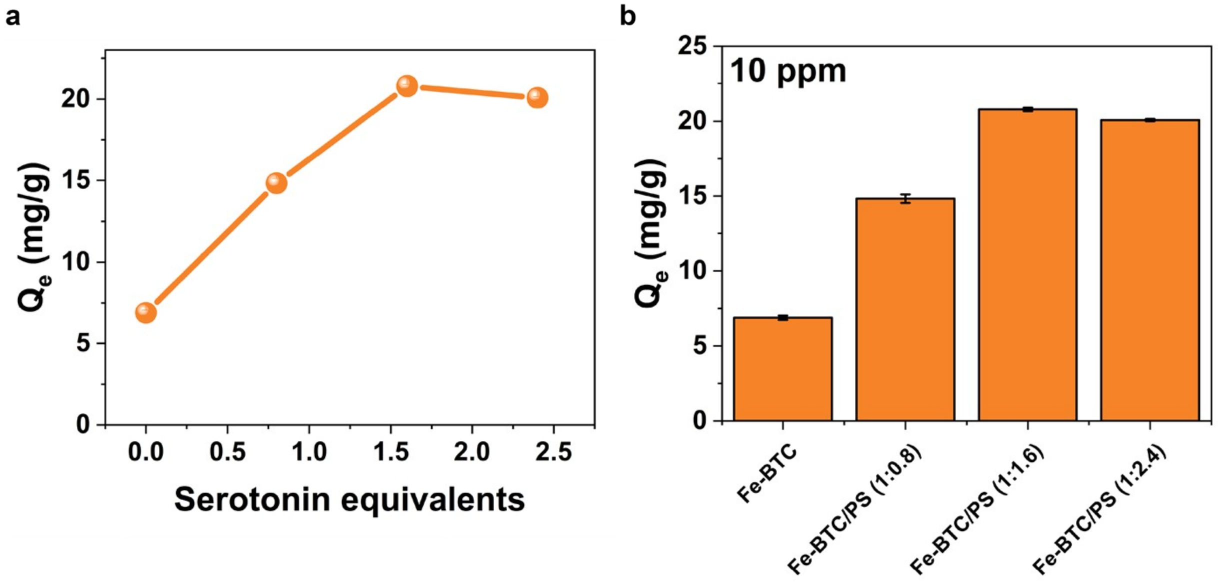

Figure S 6 Screening of polymer loading: a and b) batch adsorption results of different serotonin equivalents used in the reaction to synthesize the material.

XPS analysis of N 1S region of Fe-BTC/PS

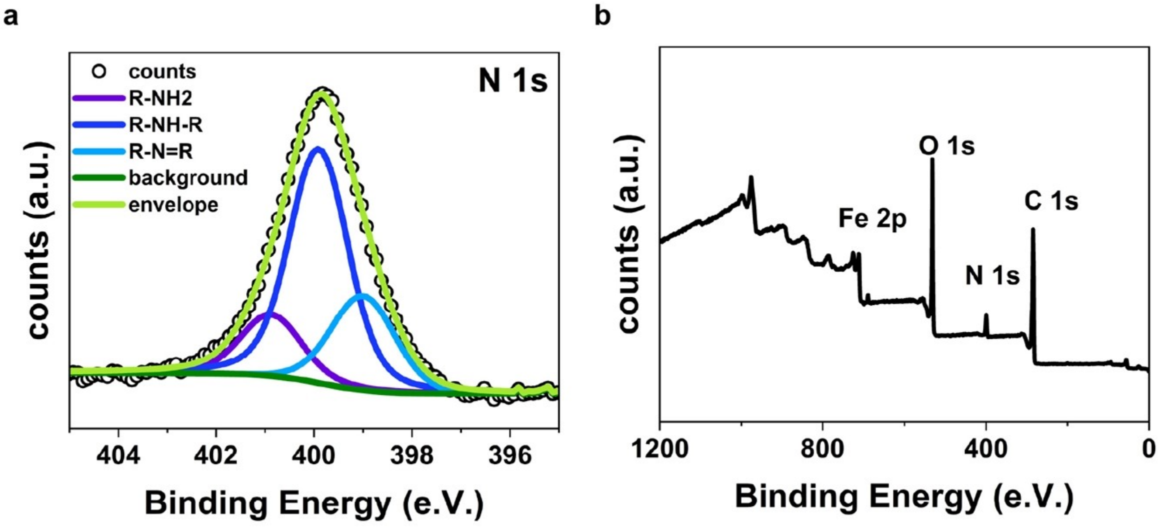

Figure S 7 Analysis of N 1S region of Fe-BTC/PS: a) Fitted nitrogen 1S region. b) Plot of wide scan spectrum.

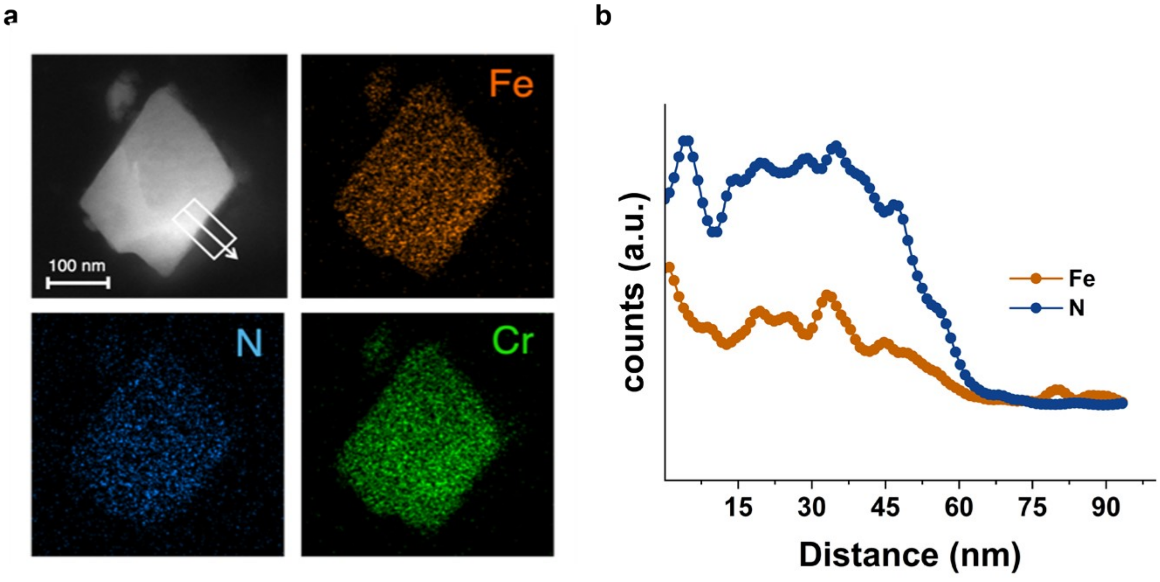

Figure S 8 STEM-EDX analysis of a sliced crystallite of Fe-BTC/PS. a) STEM images and corresponding EDX elemental maps, b) EDX intensity line scans from the region shown in a.

Adsorption isotherm pH = 3

The Cr(VI) adsorption isotherm for Fe-BTC and Fe-BTC/PS was fitted using Langmuir and Freundlich Model. The resulting fitting parameters are presented in **Table S3**.

Table S 3 Fitting parameters from Langmuir and Freundlich Model.

	Langmuir Model			Freundlich Model		
**Material**	**q_m (mg/g)**	**b**	**R^2**	**K_F**	**1/n**	**R^2**
Fe-BTC	9.80 ± 0.22	0.580 ± 0.062	0.995	11.32 ± 2.50	0.535 ± 0.06	0.961
Fe-BTC/PS	106.60 ± 5.21	0.073 ± 0.009	0.994	3.86 ± 0.55	0.260 ± 0.04	0.897

XPS analysis of Cr adsorbed by Fe-BTC/PS

Figure S 9 shows the fitting of chromium $2p_{3/2}$ spectra for the Fe-BTC/PS composite soaked in Cr(VI) solution (5 mg in a 20 mL 20 ppm solution at pH 3). It reveals that the majority of adsorbed chromium is reduced to Cr(III) (**Figure S9**). Note that the peak contribution of Cr(VI) in **Figure S 9b**, likely comes from different Cr(III) speciation. Therefore, all adsorbed chromium is likely present as Cr(III).

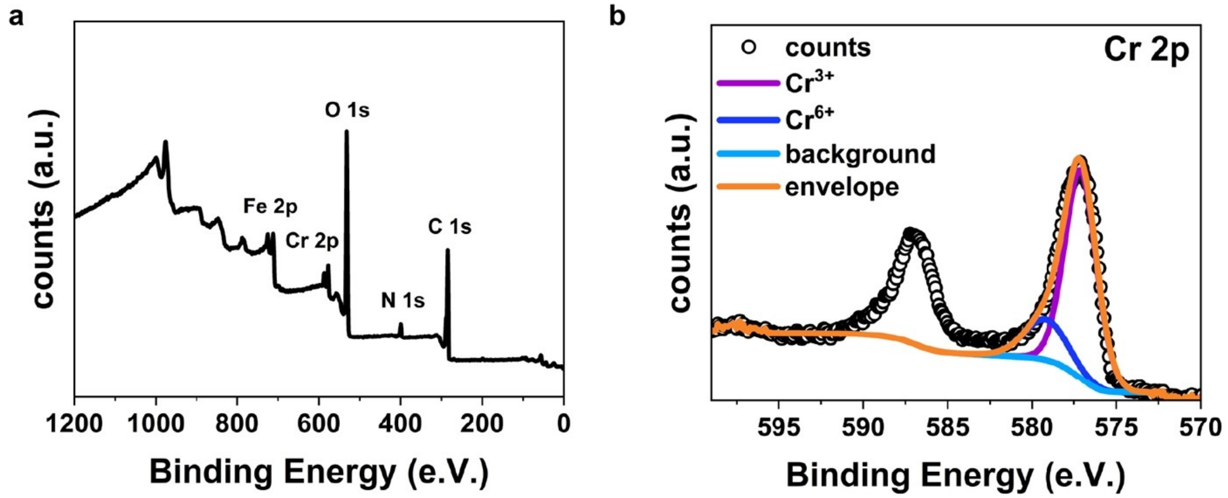

Figure S 9 XPS analysis of Fe-BTC/PS after exposure to 20 ppm Cr(VI) solution: a) wide scan. b) Chromium 2p region including fitted chromium $2p_{3/2}$ spectra for Cr(III) and Cr(VI) speciation.

Cr(III) adsorption experiment

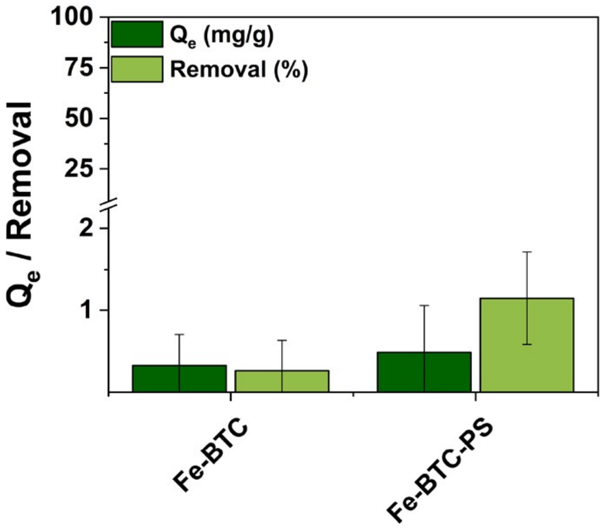

Figure S 10 Calculated Q_e (mg/g) and removal (%) of Cr(III) batch adsorption experiments for Fe-BTC and Fe-BTC/PS. Conditions; adsorption dosage = 0.25g/L, adsorption time = 24 hours, pH =3, and initial Cr concentration = 20 ppm.

Kinetic performance of the materials at pH = 3

Figure S11 shows the adsorption behavior for adsorption times up to 3 days.

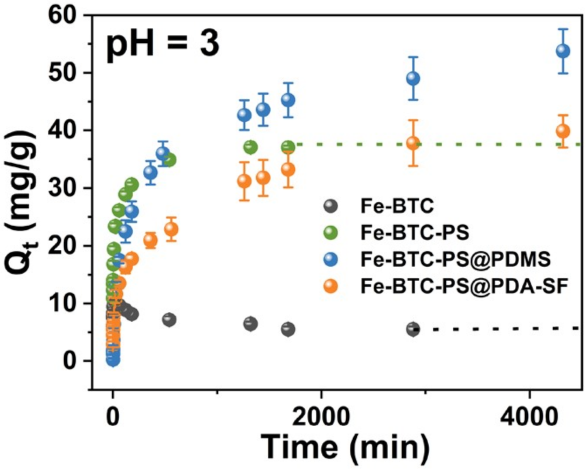

Figure S 11 Kinetic plots for Fe-BTC (black), Fe-BTC/PS (green), Fe-BTC/PS@PDA-SF (orange) and Fe-BTC/PS@PDMS (blue) for long adsorption times (3 days). Initial chromium concentration: 20ppm, pH 3, adsorbent dosage = 0.25 g/L.

Fitted adsorption experiment using pseudo second order model

Kinetic adsorption of Fe-BTC/PS, Fe-BTC/PS@PDA-SF and Fe-BTC/PS@PDMS was fitted using PSO (pseudo second order) model. The plots and linearized fitting are presented in **Figure S12**.

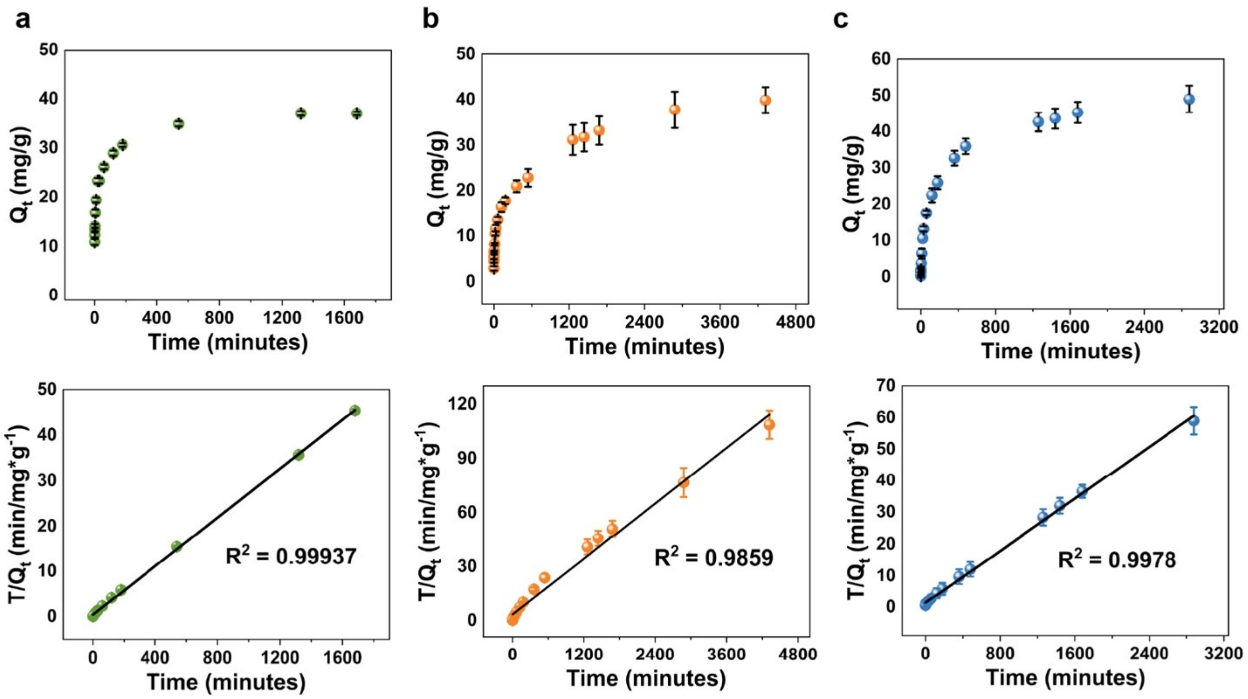

Figure S 12 Kinetic adsorption plots and linear fit for a) Fe-BTC/PS (green), b) Fe-BTC@PDA-SF (orange) and c) Fe-BTC/PS@PDMS (blue).

Table S 4 Parameters obtained by fitting the pseudo second-order-model

Material	k_2 (g/(mg min))	q_e (mg/g)	R^2
Fe-BTC/PS	0.0018	37.231	0.9993
Fe-BTC/PS@PDMS	$3.057*10^{-4}$	48.638	0.9978
Fe-BTC/PS@PDA-SF	$1.933*10^{-4}$	38.933	0.9859

Stability of Fe-BTC, Fe-BTC/PS and coated hydrophobic materials in acidic conditions

Figure S13a shows the amount of iron (Fe) leached into solution. **Figure S13b** presents the calculated percentage of Fe leached relative to the total iron content in the MOF. The total iron content was calculated based on TGA data of the material, using the residual weight percentage at 800 °C.

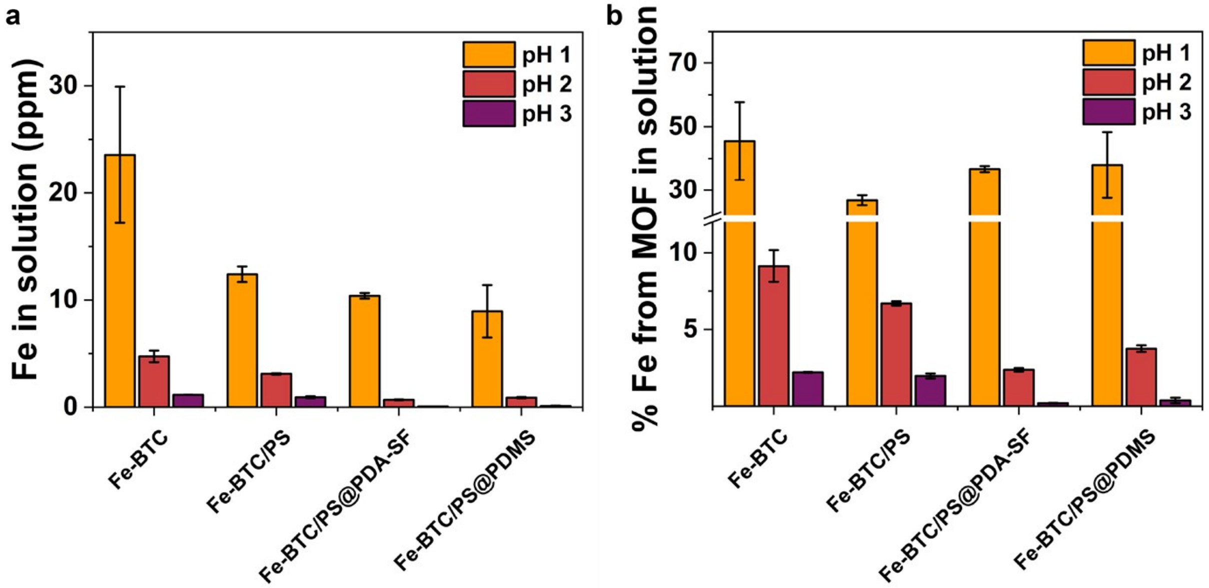

Figure S 13 Stability analysis of the materials: a) leached Fe into solution at pH 1 (orange), pH 2 (red) and pH 3 (purple). b) Fe leached relative to the total iron content in used MOF and MOF/polymer composite.

Performance of Fe-BTC, Fe-BTC/PS and coated hydrophobic materials in acidic conditions

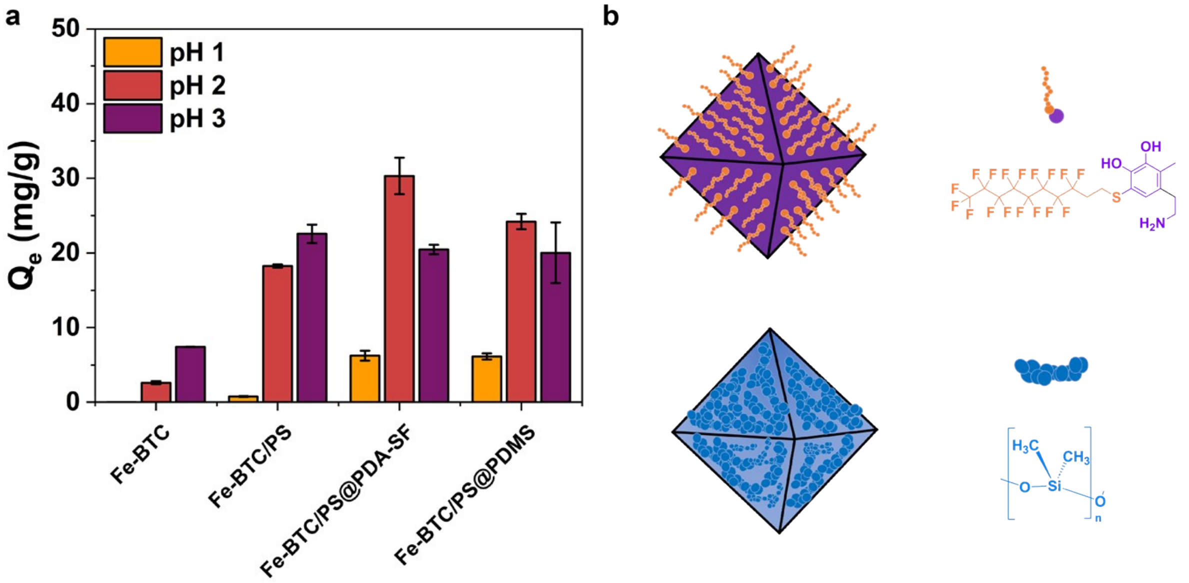

Figure S 14 Overview batch adsorption experiment of Fe-BTC, Fe-BTC/PS and coated materials Fe-BTC/PS and Fe-BTC/PS@PDA-SF, respectively in acid conditions. a) Removal capacities at different pH with adsorption dosage = 0.25g/L, adsorption time = 24 hours and initial chromium concentration of 10 ppm. b) Schematic representation of the two coated materials.

Characterization coated MOF/polymer composites

Fe-BTC/PS-@PDA-SF

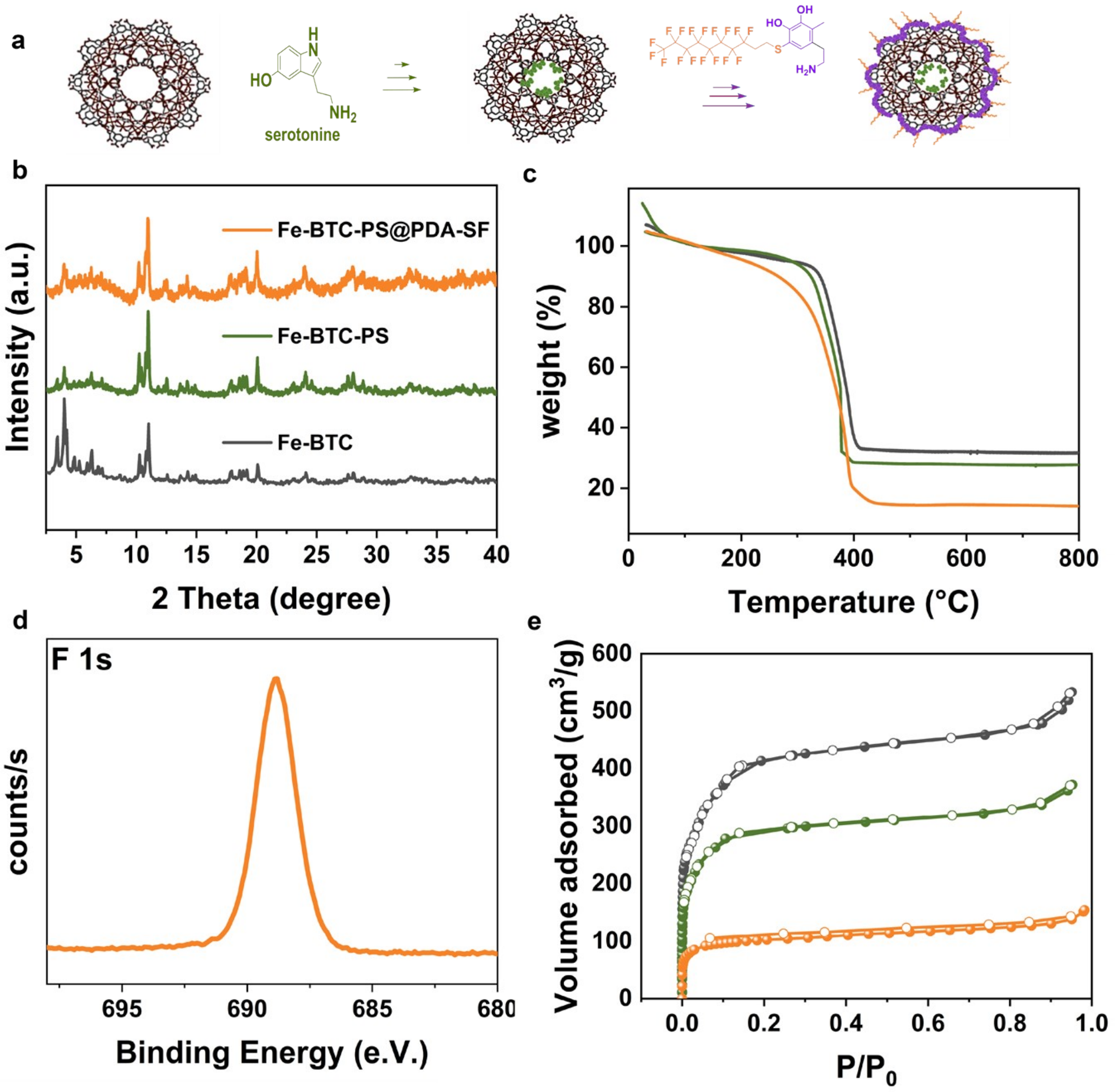

Figure S 15 Characterization of perfluorodecanethiol@PDA coated Fe-BTC/PS, a) Schematic representation of reaction to Fe-BTC, Fe-BTC/PS and coated Fe-BTC/PS-@PDA-SF. b) Powder X-ray diffraction patterns of the Fe-BTC (black), Fe-BTC/PS (green) and Fe-BTC/PS@PDA-SF (orange). c) Thermogravimetric analysis curves. d) XPS F 1s region of Fe-BTC/PS-@PDA-SF. e) N_2 adsorption isotherms

Fe-BTC/PS@PDMS

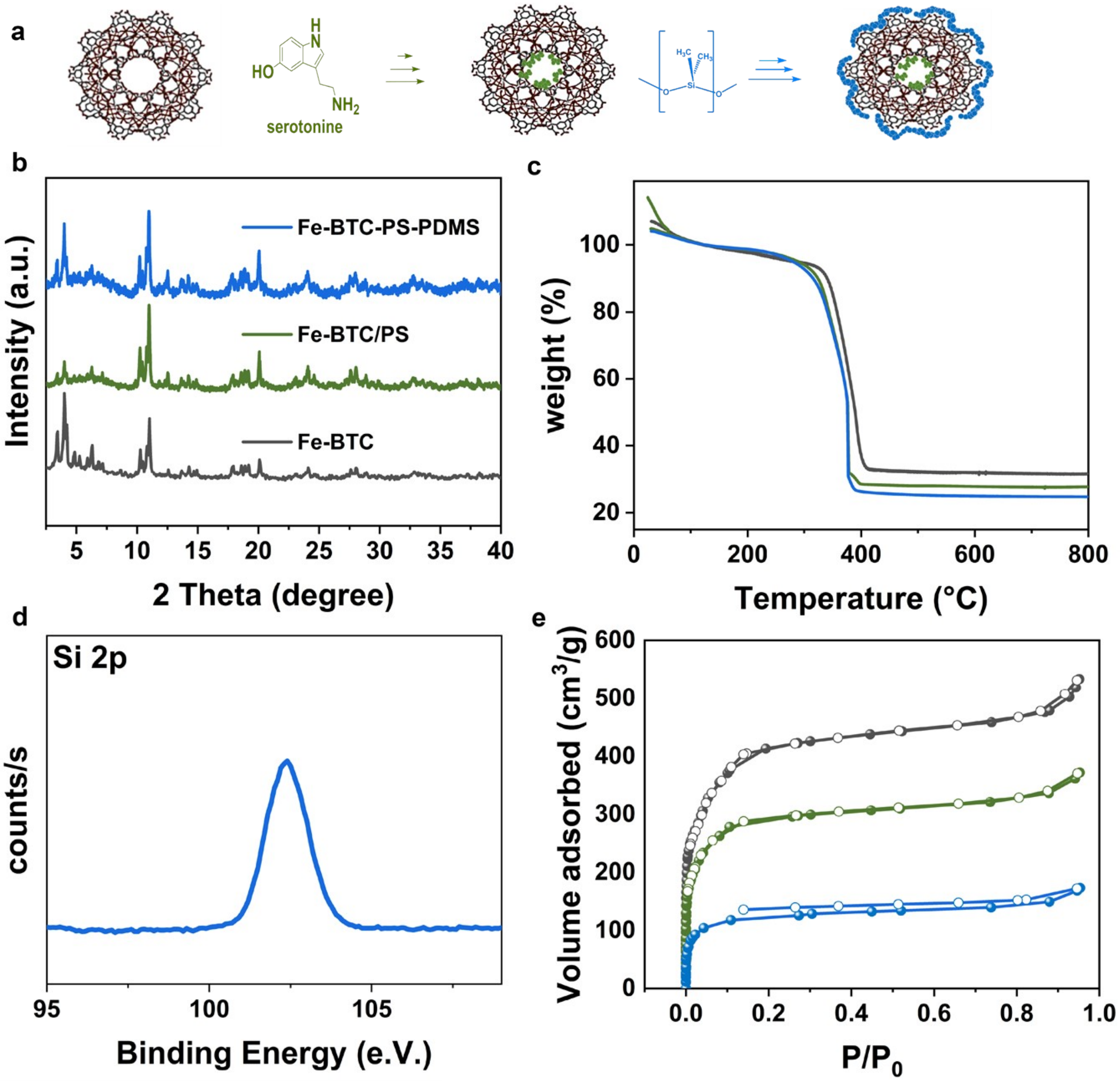

Figure S 16 Characterization of PDMS coated Fe-BTC/PS: a) Schematic representation of reaction to Fe-BTC/PS and coated Fe-BTC/PS-PDMS. b) Powder X-ray diffraction patterns of the Fe-BTC (black), Fe-BTC/PS (green) and Fe-BTC/PS-PDMS (blue). c) Thermogravimetric analysis curves. d) XPS Si 2p region of Fe-BTC/PS/PDMS. e) N_2 adsorption isotherms.

STEM-EDX analysis of coated materials Fe-BTC/PS@PDA-SF

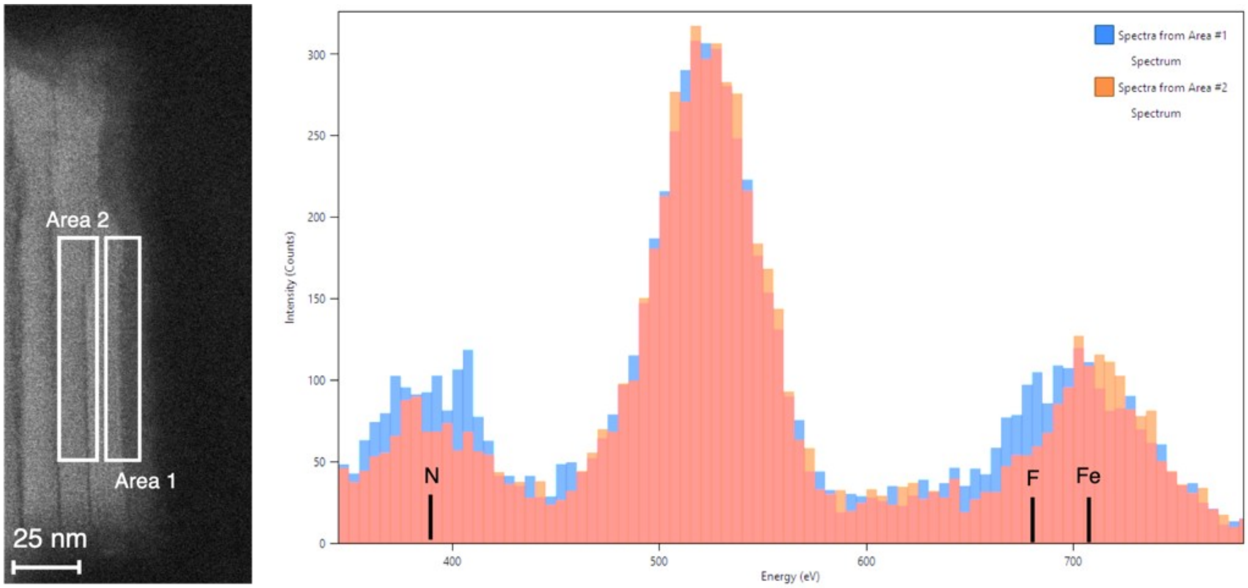

Figure S 17 STEM image of a sliced crystallite of Fe-BTC/PS@PDA-SF along with the corresponding EDX spectra from two distinct regions: one on the outer surface of the crystallite (area 1), and the other from the interior of the crystallite (area 2).

Contact angle Fe-BTC/PS@PDMS and Fe-BTC/PS@PDA-SF

Figure S18 shows the image of the contact angle measurement which is also summarized in **Table S5**.

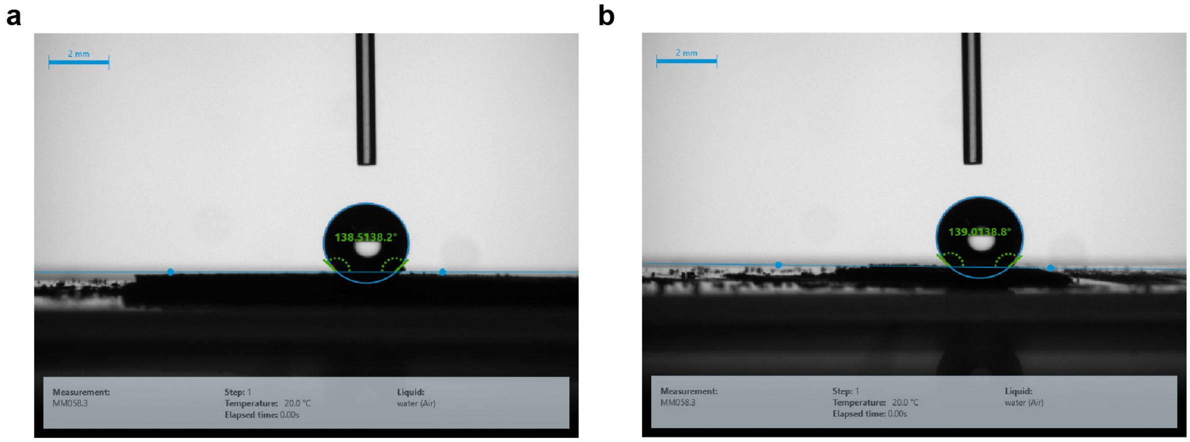

Figure S 18 Photograph of contact angle measurement of a) Fe-BTC/PS@PDMS and b) Fe-BTC/PS@PDA-SF

Table S 5 Results contact angle measurement

Material	Solvent	Temperature	Contact angle
Fe-BTC/PS@PDMS	H_2O	20°C	138.51°
Fe-BTC/PS@PDA-SF	H_2O	20°C	139.01°

PXRD analysis of Fe-BTC, Fe-BTC/PS, and coated materials in acidic conditions

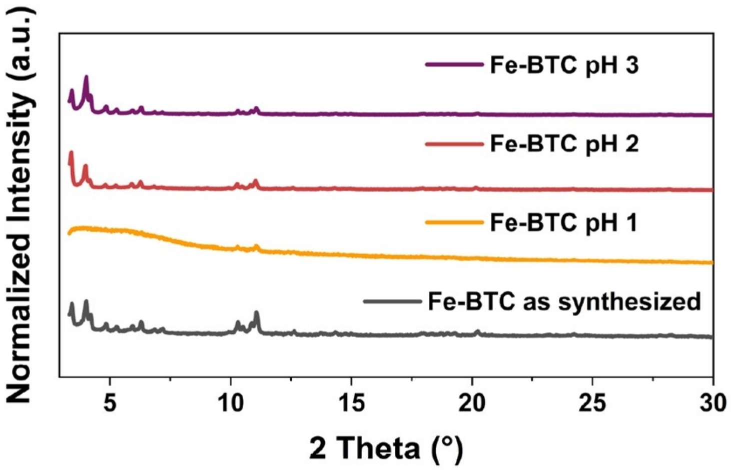

Figure S 19 PXRD of Fe-BTC after Cr(VI) adsorption experiments at different pH; adsorption dosage = 0.25g/L, adsorption time = 24 hours and initial chromium concentration of 20 ppm.

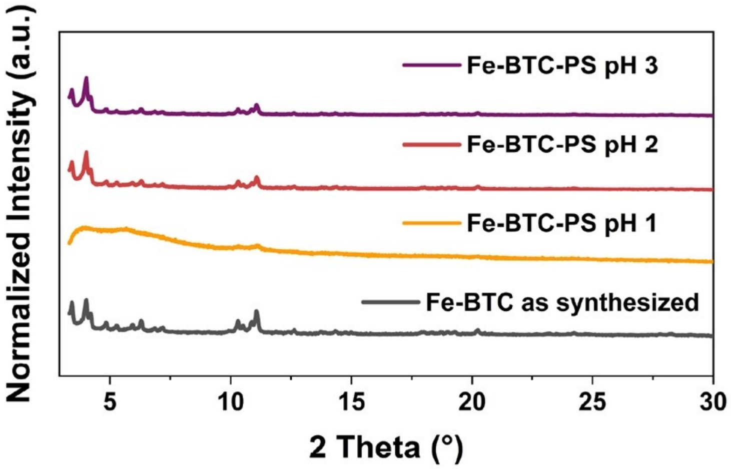

Figure S 20 PXRD of Fe-BTC/PS after Cr(VI) adsorption experiments at different pH; adsorption dosage = 0.25g/L, adsorption time = 24 hours and initial chromium concentration of 20 ppm.

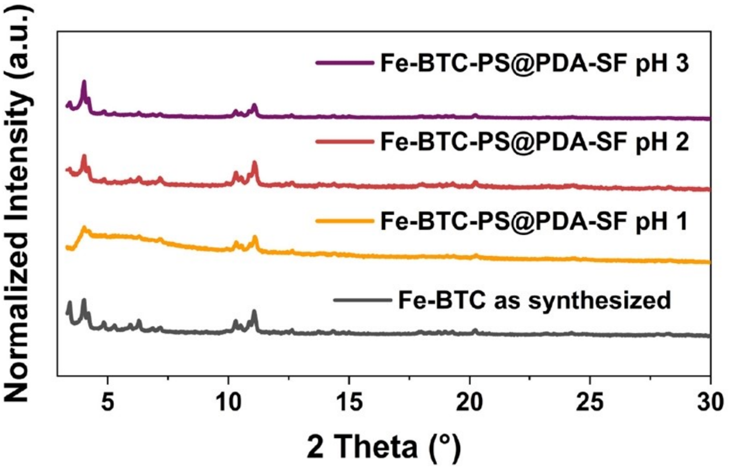

Figure S 21 PXRD of Fe-BTC/PS@PDA-SF after Cr(VI) adsorption experiments at different pH; adsorption dosage = 0.25g/L, adsorption time = 24 hours and initial chromium concentration of 20 ppm.

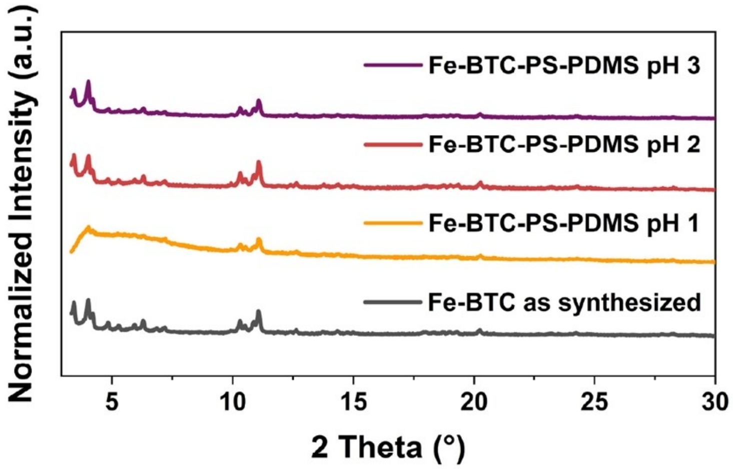

Figure S 22 PXRD of Fe-BTC/PS@PDMS after Cr(VI) adsorption experiments at different pH; adsorption dosage = 0.25g/L, adsorption time = 24 hours and initial chromium concentration of 20 ppm.

Characterization different metal-based MOF/polymer

Zr-BDC/PS

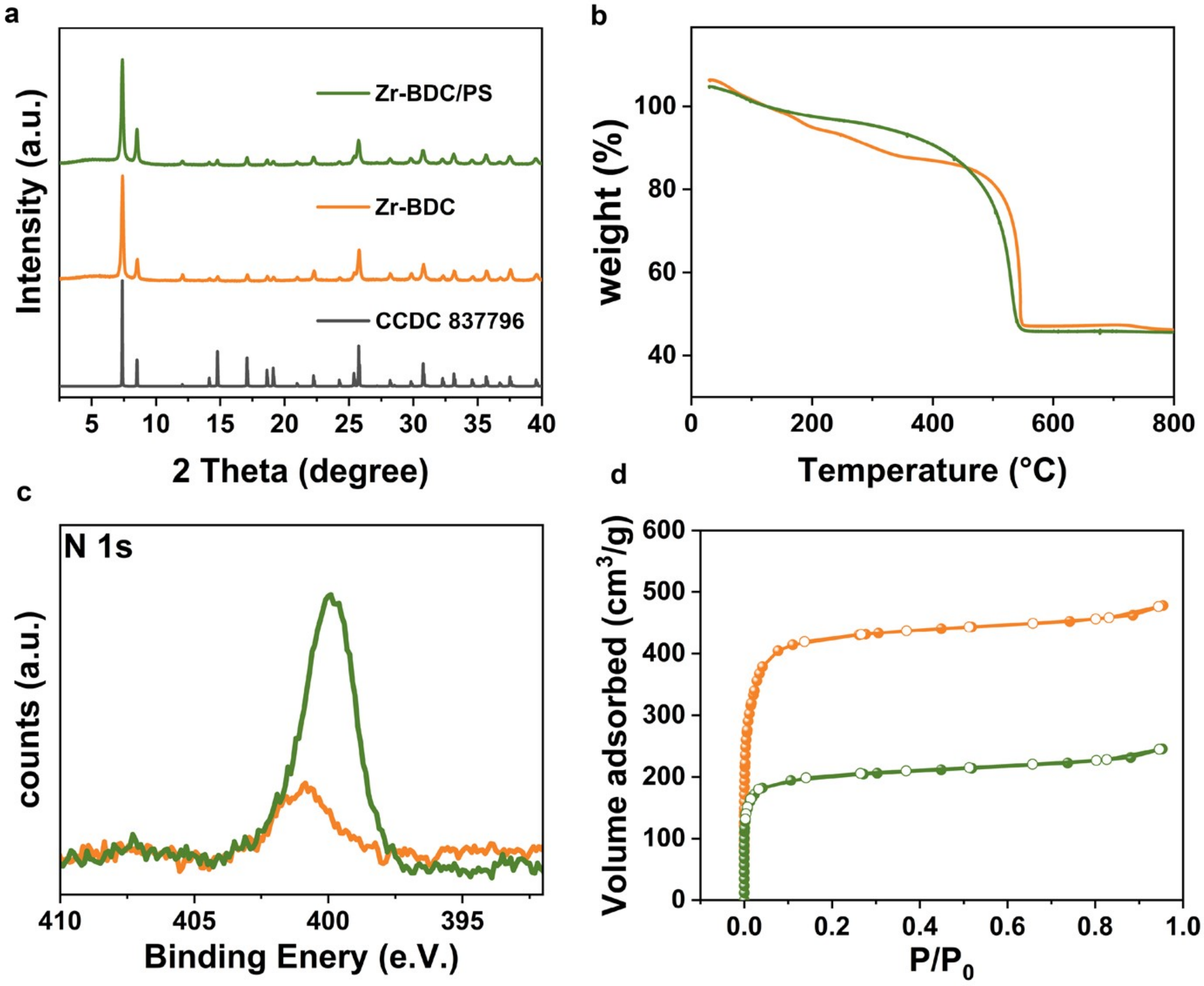

Figure S 23 Characterization of Zr-BDC-PS: a) powder X-ray diffraction patterns of simulated Zr-BDC (black), as-synthesized Zr-BDC (orange) and Zr-BDC/PS (green), b) thermogravimetric analysis curves, c) XPS N 1S region of Zr-BDC (orange) and Zr-BDC/PS (green). d) N_2 adsorption isotherms

Cr-BDC-PS

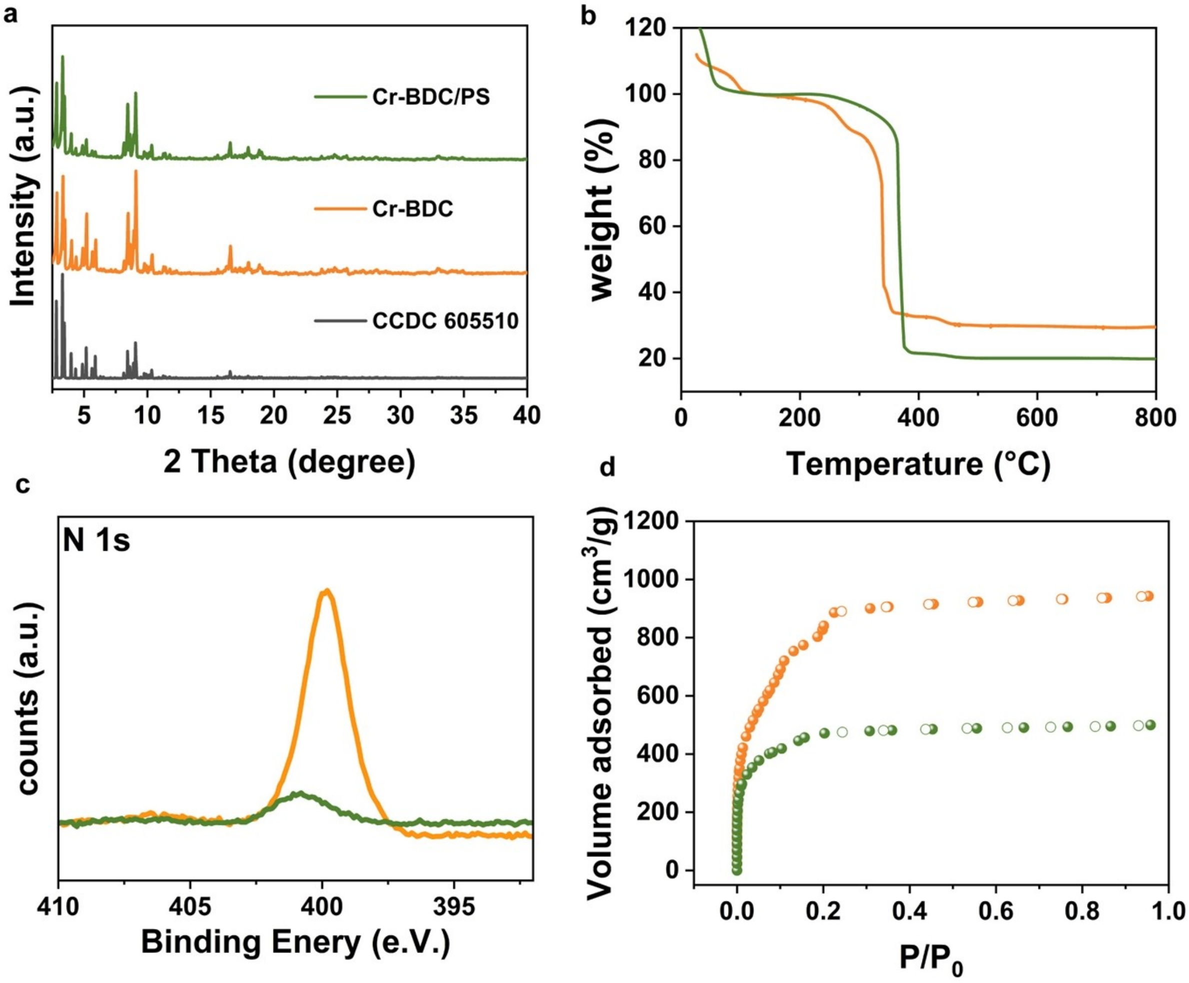

Figure S 24 Characterization of Cr-BDC-PS: a) Powder X-ray diffraction patterns of simulated Cr-BDC (black), as-synthesized Cr-BDC (orange) and Cr-BDC/PS (green). b) Thermogravimetric analysis curves. c) XPS N 1S region. d) N_2 adsorption isotherms

Performance of other MOF-PS composites in acidic conditions

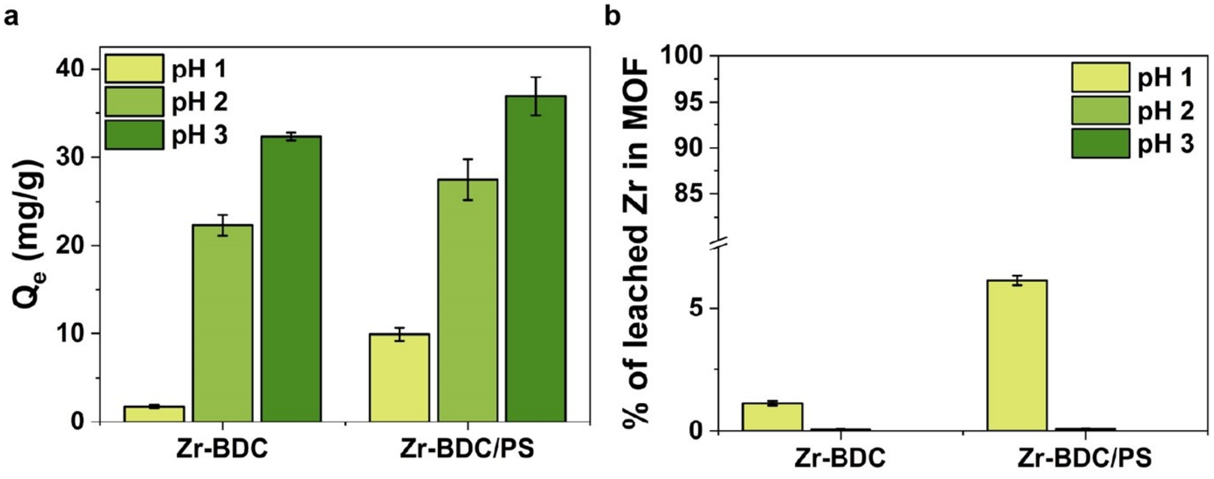

Figure S 25 Cr(VI) adsorption performance and metal leaching of Zr-BDC and Zr-BDC-PS at different pH: a) calculated Q_e of Cr(VI) batch adsorption experiments; conditions; adsorption dosage = 0.25g/L, adsorption time = 24 hours, initial Cr concentration = 20 ppm, pH 3, 2 and 1, respectively, b) calculated % of Zr from MOF leached in solution during batch adsorption experiments measured via ICP-OES.

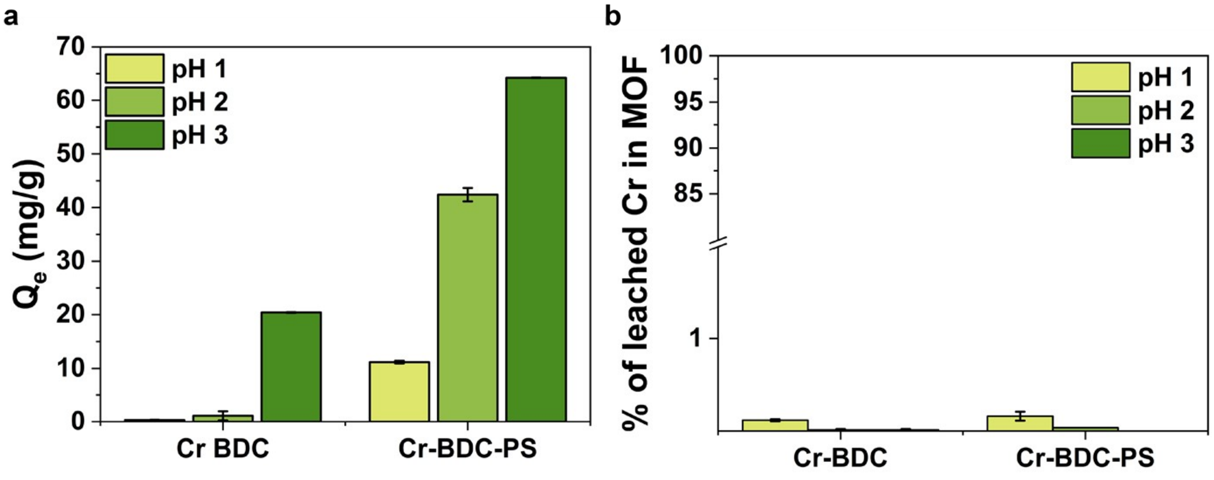

Figure S 26 Cr(VI) adsorption performance and metal leaching in MilliQ water for of Cr-BDC and Cr-BDC-PS at different pH: a) calculated Q_e of Cr(VI) batch adsorption experiments; conditions; adsorption dosage = 0.25g/L, adsorption time = 24 hours, initial Cr concentration = 20 ppm, pH 3, 2 and 1, respectively, b) calculated % of Cr from MOF leached in solution during batch adsorption experiments measured via ICP-OES.

Stability of other MOF-PS composites in acidic conditions (pH 3)

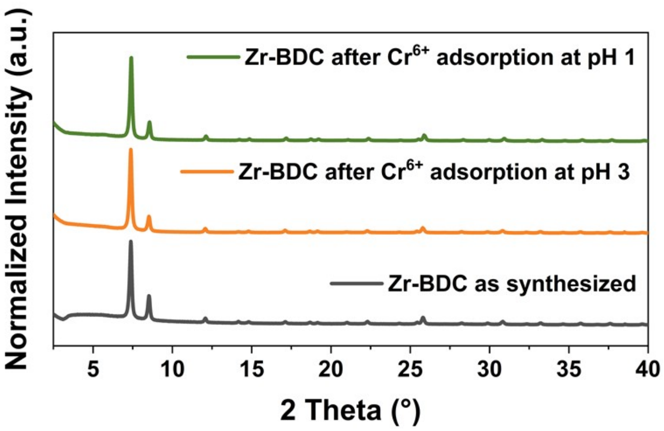

Figure S 27 PXRD of Zr-BDC after adsorption of chromium at pH 3 and pH 1.

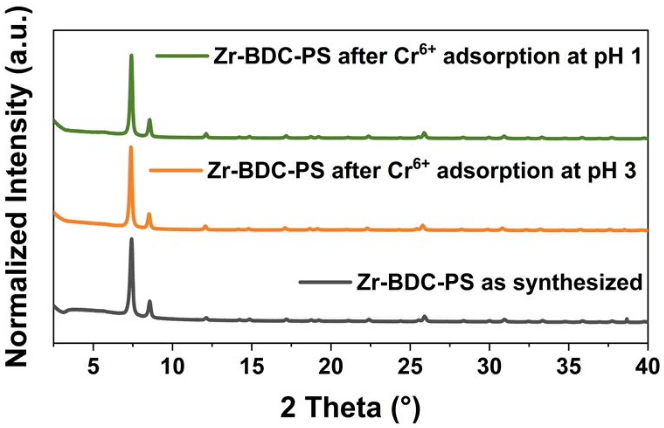

Figure S 28 PXRD of Zr-BDC/PS after adsorption of chromium at pH 3 and pH 1.

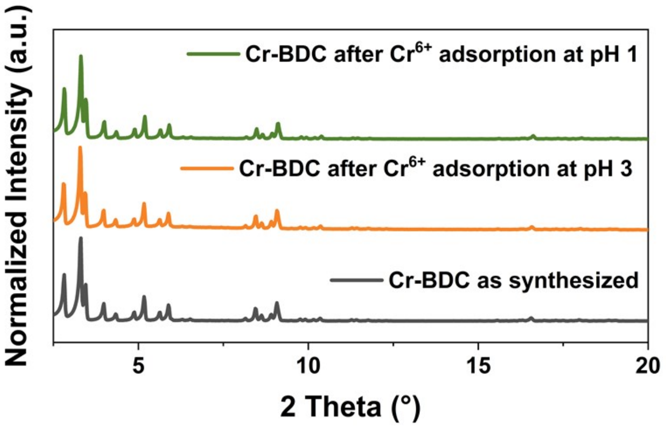

Figure S 29 PXRD of Cr-BDC after adsorption of chromium at pH 3 and pH 1.

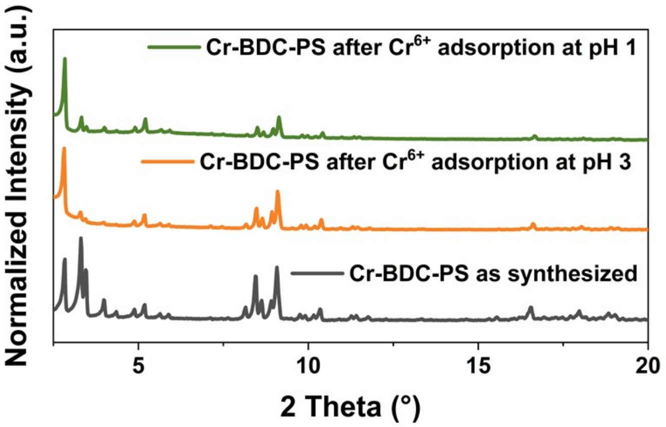

Figure S 30 PXRD of Cr-BDC/PS after adsorption of chromium at pH 3 and pH 1.

Selection of best performing material in neutral (pH=7) conditions

At pH 7, the trivalent chromium is presumably present in form of $Cr(OH)_3$ whereas the hexavalent chromium is present in the form of CrO_4^{2-}.[7] To investigate the performance of the different incorporated polymer, a screening at low initial Cr(VI) (1ppm) concentration was carried out (**Figure S31**). Similar to the observed results in acidic aqueous media (pH 3, **Figure 1**), in neutral conditions (pH 7), the MOF/polymer PpAP, PpPDA, PDA and PS outperformed the other polymers at low initial concentration (**Figure S31a**). To gain further insight into the adsorption performance of the best performing MOF/polymer composites, adsorption experiment at 10 ppm Cr(VI*)* were carried out, revealing the highest uptake of Cr(VI) from neutral solution by Fe-BTC/PS followed by Fe-BTC/PDA (**Figure S31b**). Therefore, for real river water sample, Fe-BTC/PDA and Fe-BTC/PS were investigated.

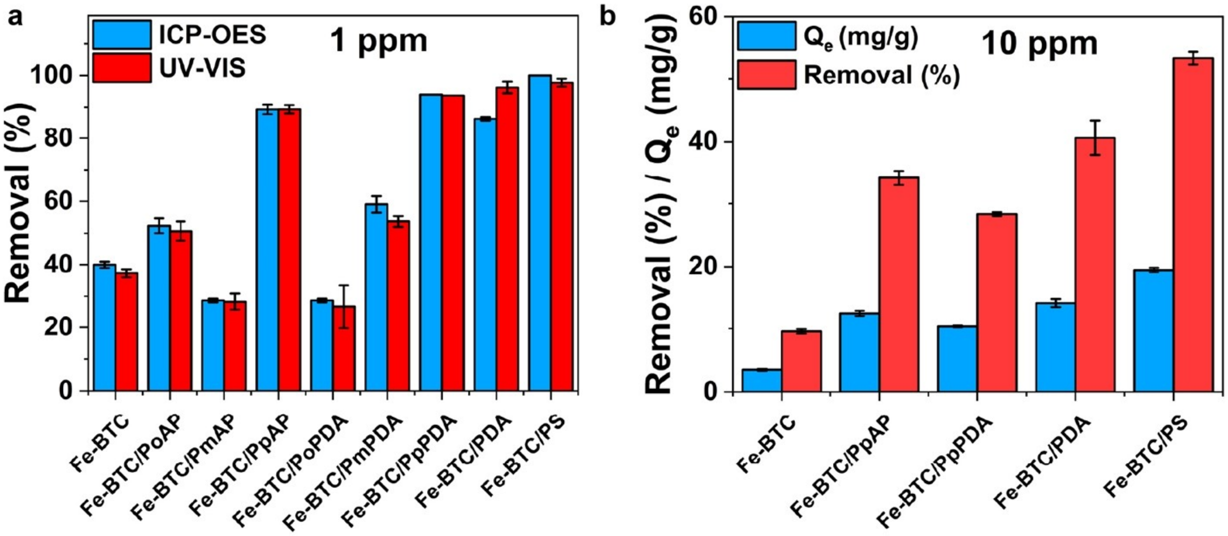

Figure S 31 Overview results batch adsorption experiment in neutral pH (pH 7): a) batch adsorption experiment analyzed by ICP-OES (blue) and UV-VIS (red) of all MOF/polymer composites, with adsorption dosage = 0.25g/L, adsorption time = 24 hours and initial chromium concentration of 1 ppm. b) batch adsorption experiment analyzed by ICP-OES of best performing materials in Figure 29a at higher initial chromium concentration of 10 ppm, adsorption dosage = 0.25g/L, adsorption time = 24 hours.

Performance of Fe-BTC/PDA and Fe-BTC/PS in real river water conditions

Figure S32 shows, the extraction of 900 ppb Cr(VI) spiked river water for different adsorption dosage (0.5 g/L, 0.25 g/L of 0.15 g/L) of Fe-BTC/PS and Fe-BTC/PDA, respectively.

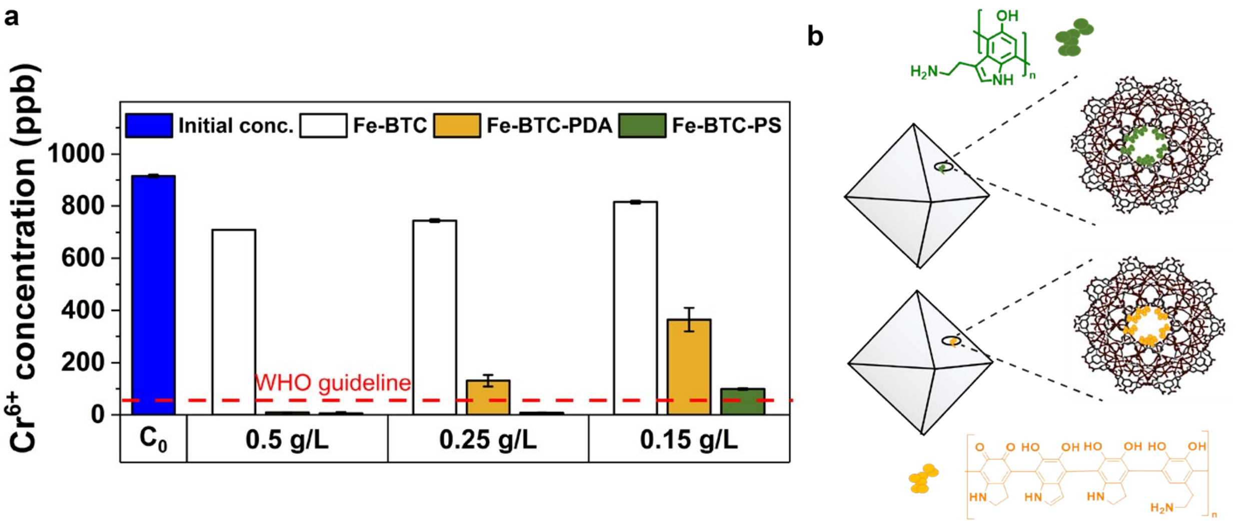

Figure S 32 Overview adsorption performance of material in real-world water sample (Rhone River water): a) Cr concentration after adsorption experiment with different adsorbent dosage, b) schematic representation of the two tested Fe-BTC-polymer – Fe-BTC/PS (green), Fe-BTC/PDA (orange).

References

1 M. J. Katz, Z. J. Brown, Y. J. Colón, P. W. Siu, K. A. Scheidt, R. Q. Snurr, J. T. Hupp and O. K. Farha, *Chem. Commun.*, 2013, **49**, 9449.

2 L. Peng, S. Yang, D. T. Sun, M. Asgari and W. L. Queen, *Chem. Commun.*, 2018, **54**, 10602–10605.

3 S. Yang, L. Peng, D. T. Sun, M. Asgari, E. Oveisi, O. Trukhina, S. Bulut, A. Jamali and W. L. Queen, *Chem. Sci.*, 2019, **10**, 4542–4549.

4 M. Long, S. Peng, W. Deng, X. Miao, N. Wen, Q. Zhou, X. Yang and W. Deng, *J. Mater. Chem. A*, 2017, **5**, 22761–22771.

5 J. W. M. Osterrieth, J. Rampersad, D. Madden, N. Rampal, L. Skoric, B. Connolly, M. D. Allendorf, V. Stavila, J. L. Snider, R. Ameloot, J. Marreiros, C. Ania, D. Azevedo, E. Vilarrasa-Garcia, B. F. Santos, X. Bu, Z. Chang, H. Bunzen, N. R. Champness, S. L. Griffin, B. Chen, R. Lin, B. Coasne, S. Cohen, J. C. Moreton, Y. J. Colón, L. Chen, R. Clowes, F. Coudert, Y. Cui, B. Hou, D. M. D’Alessandro, P. W. Doheny, M. Dincă, C. Sun, C. Doonan, M. T. Huxley, J. D. Evans, P. Falcaro, R. Ricco, O. Farha, K. B. Idrees, T. Islamoglu, P. Feng, H. Yang, R. S. Forgan, D. Bara, S. Furukawa, E. Sanchez, J. Gascon, S. Telalović, S. K. Ghosh, S. Mukherjee, M. R. Hill, M. M. Sadiq, P. Horcajada, P. Salcedo-Abraira, K. Kaneko, R. Kukobat, J. Kenvin, S. Keskin, S. Kitagawa, K. Otake, R. P. Lively, S. J. A. DeWitt, P. Llewellyn, B. V. Lotsch, S. T. Emmerling, A. M. Pütz, C. Martí-Gastaldo, N. M. Padial, J. García-Martínez, N. Linares, D. Maspoch, J. A. Suárez Del Pino, P. Moghadam, R. Oktavian, R. E. Morris, P. S. Wheatley, J. Navarro, C. Petit, D. Danaci, M. J. Rosseinsky, A. P. Katsoulidis, M. Schröder, X. Han, S. Yang, C. Serre, G. Mouchaham, D. S. Sholl, R. Thyagarajan, D. Siderius, R. Q. Snurr, R. B. Goncalves, S. Telfer, S. J. Lee, V. P. Ting, J. L. Rowlandson, T. Uemura, T. Iiyuka, M. A. Van Der Veen, D. Rega, V. Van Speybroeck, S. M. J. Rogge, A. Lamaire, K. S. Walton, L. W. Bingel, S. Wuttke, J. Andreo, O. Yaghi, B. Zhang, C. T. Yavuz, T. S. Nguyen, F. Zamora, C. Montoro, H. Zhou, A. Kirchon and D. Fairen-Jimenez, *Advanced Materials*, 2022, **34**, 2201502.

6 Z. A. Zakaria, Z. Zakaria, S. Surif and W. A. Ahmad, *Journal of Hazardous Materials*, 2007, **146**, 30–38.

7 D. Rai, L. E. Eary and J. M. Zachara, *Science of The Total Environment*, 1989, **86**, 15–23.
